# Highly Efficient Near-Infrared Light-Driven Molecular Motor Rotation Enabled by Upconversion Nanoparticles as Nanoscale Light Sources

**DOI:** 10.1021/jacs.5c07953

**Published:** 2025-07-17

**Authors:** Jinyu Sheng, Youxin Fu, Kefan Wu, Thomas Freese, Hong Zhang, Ben L. Feringa

**Affiliations:** † Stratingh Institute for Chemistry, 3647 University of Groningen, Nijenborgh 3, Groningen 9747 AG, The Netherlands; ‡ College of Chemistry, Chemical Engineering and Materials Science, Soochow University, Suzhou, Jiangsu 215123, China; § College of Science, Nanjing Forestry University, Nanjing 210037, P.R. China; ∥ Van’t Hoff Institute for Molecular Sciences, 1234 University of Amsterdam, Science Park 904, Amsterdam 1098 XH, The Netherlands

## Abstract

Powering light-driven molecular motors with visible or near-infrared (NIR) light is essential in the design of molecular machines, bringing dynamic functions to the next generation of responsive materials particularly for biological applications. However, current strategies suffer from heavy molecular substitution and low photoefficiency of excitation, limiting their practical use in bulk materials and biomolecular systems. Here, we report a general and highly efficient strategy to power NIR light-driven molecular motors via a radiative energy transfer mechanism. Taking advantage of spectrally tunable upconversion nanoparticles (UCNPs), the motors powered by continuous wave NIR light can reach photostationary states (PSS) with high efficiency, comparable to those of direct UV/visible light-driven systems, without a deaeration process needed. The concept is validated on various molecular motors with different rotary speeds, providing a general, broadly applicable principle for the future design of highly efficient NIR-powered photodynamic molecular motor systems.

## Graphical abstract

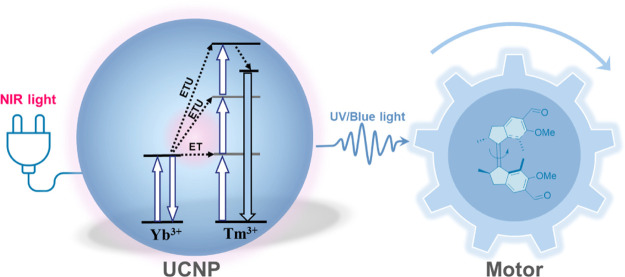

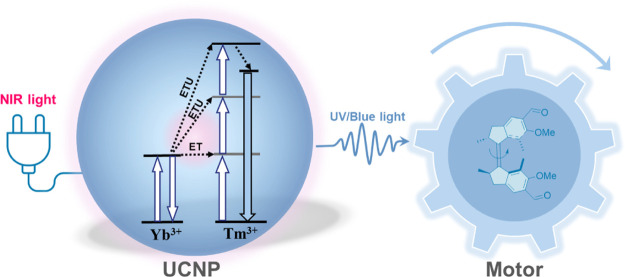

## Introduction

Natural molecular motors play a central role in driving living systems out of equilibrium in order to fulfill a wide range of biological functions. Inspired by these fascinating nanomachines, artificial molecular machines (AMMs)^1–9^ have attracted great interests in the past two decades and exhibit tremendous potential toward dynamic hybrid molecular systems as well as mechanically responsive (bio)­materials.^10–15^ As a unique class of AMMs, photochemically powered overcrowded alkene-derived rotary molecular motors^16^ (and switches)^17,18^ can harvest light energy to achieve unidirectional rotary motion controlled by their intrinsic chirality, which renders them highly attractive in modulation of dynamic functions with noninvasive input.^19,20^ The detrimental ultraviolet (UV) light activation of molecular motors (Figure 1A,B)^21–25^ is arguably to be overcome for a number of applications especially in biomedical applications.^26,27^ To address this challenge, approaches have been developed to bathochromically shift the absorption wavelength of motors into the visible region by π-system extension,^28–30^ inducing push–pull substitution^31–33^ and metal complexation (Figure 1A).^34–37^ The construction of new scaffolds such as oxindole^38^ and hemithioindigo-derived^39,40^ molecular motors responding to visible light meanwhile provides opportunities toward future applications.^15^ However, many applications—particularly in biological systems and bulk materials—require further red-shifting of the excitation wavelengths to achieve deeper penetration and minimize phototoxicity. Recently, by covalent attachment of a photosensitizer, a two-photon absorption (2PA) strategy has shown to be successful in excitation of the second-generation molecular motors using 800 nm near-infrared (NIR) light involving a resonance energy transfer (RET) mechanism (Figure 1B).^41^ Though such strategies are promising, most of these motors require specific substitutions, which impede further functionalization. Furthermore, the low efficiency in powering molecular motors applying the pulsed 2PA approach severely hinders practical applications, combined with high intensity NIR light which will potentially decompose organic molecules.^13^

**Figure 1:**
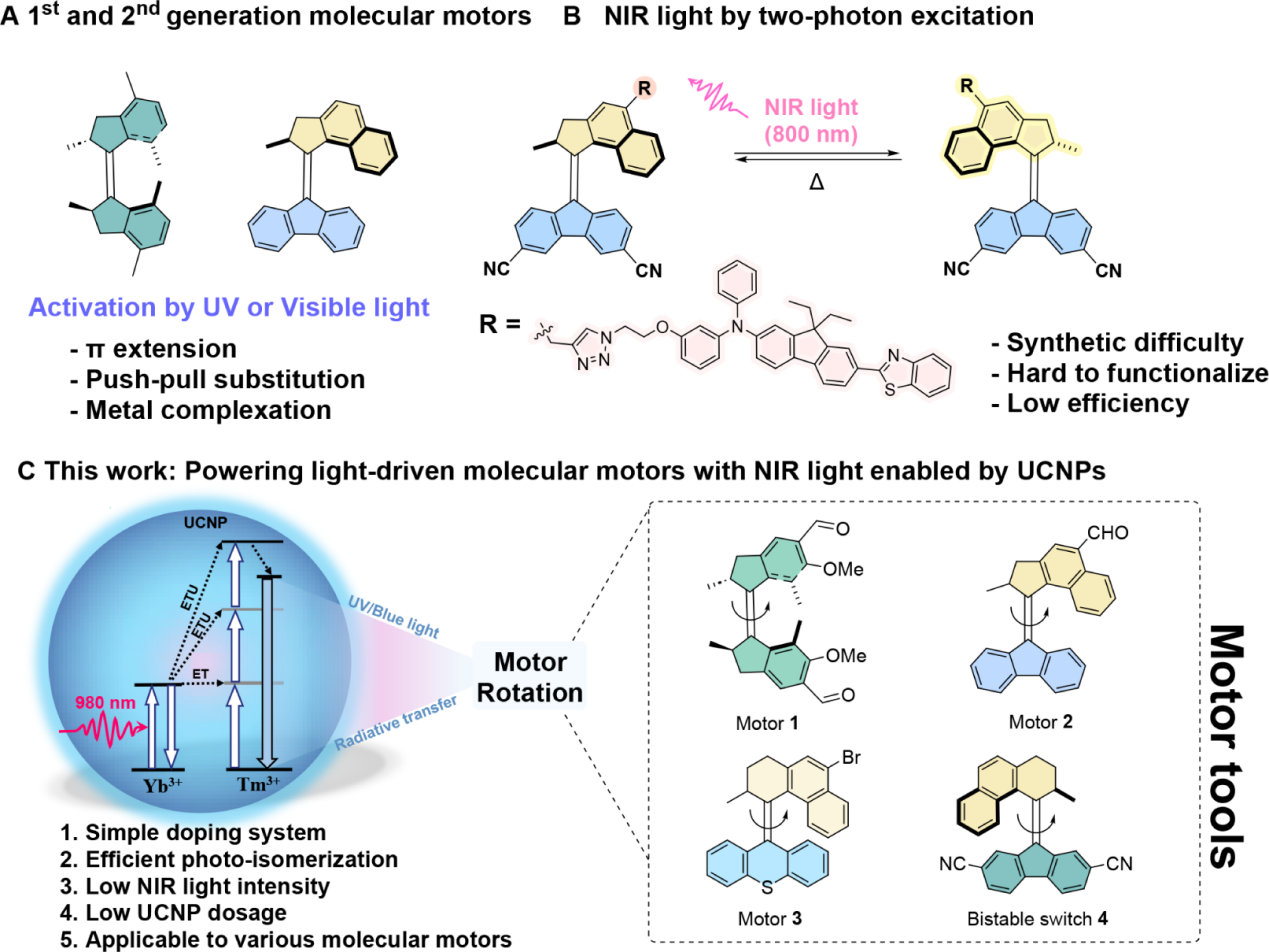
Overview of molecular motors powered by light. (A) UV/visible-light-driven molecular motors; (B) NIR light powered second-generation molecular motors via a two-photon excitation mechanism; (C) General strategy to power rotary molecular motors and switches by NIR-light through UCNPs.

Therefore, there is a great demand for a practical and universal indirect-excitation strategy^42,43^ to trigger motor rotation in an efficient and robust manner without fatigue under NIR light, without compromising the stability and functionality of the motor structures, which could be ideally applied in biological applications for the future.

Lanthanide-doped upconversion nanoparticles (UCNPs)^44–46^ capable of converting low-energy NIR light to high-energy UV and visible light have attracted much attention in triggering photoreactions by NIR light. Owing to their excellent photophysical properties including long emission lifetimes, narrow emission bandwidths, tunable emission spectra, and high photostability, UCNPs have seen widespread applications ranging from bioimaging and sensors to photovoltaics, solid-state devices.^47–50^ More importantly, the wide use of UCNPs in theranostics indicates negligible toxicity.^51^ Thus, utilizing this strategy to power light-driven rotary molecular motors by NIR light enabled by UCNPs is undoubtedly highly appealing, particularly in mimicking biological molecular machines and functional material systems. However, successful reports have yet to emerge, likely due to the lack of suitable motor candidates that were envisioned before.^41^ Here, we present a general and practical approach to powering light-driven molecular motors using 980 nm NIR light through the radiative energy transfer of the UCNPs emitted photons (Figure 1C), exhibiting the highest efficiency and photoconversion yields among all indirect strategies.

In this study, doped NaYF_4_ nanoparticles were selected for light upconversion due to their excellent performance in photochemical energy conversion and robustness.^52^ Transmission electron microscopy (TEM) images depict the hexagonal-shaped morphology of the nanoparticles with high uniformity in size (Figure 2A, for detailed information, see Section S2, Figure S2). Upon 980 nm laser excitation, the UCNPs show a wide range of emission, i.e., 340, 360, 450, and 470 nm (Figure 2B), respectively, which are suitable wavelengths for activating widely used molecular motors.

**Figure 2:**
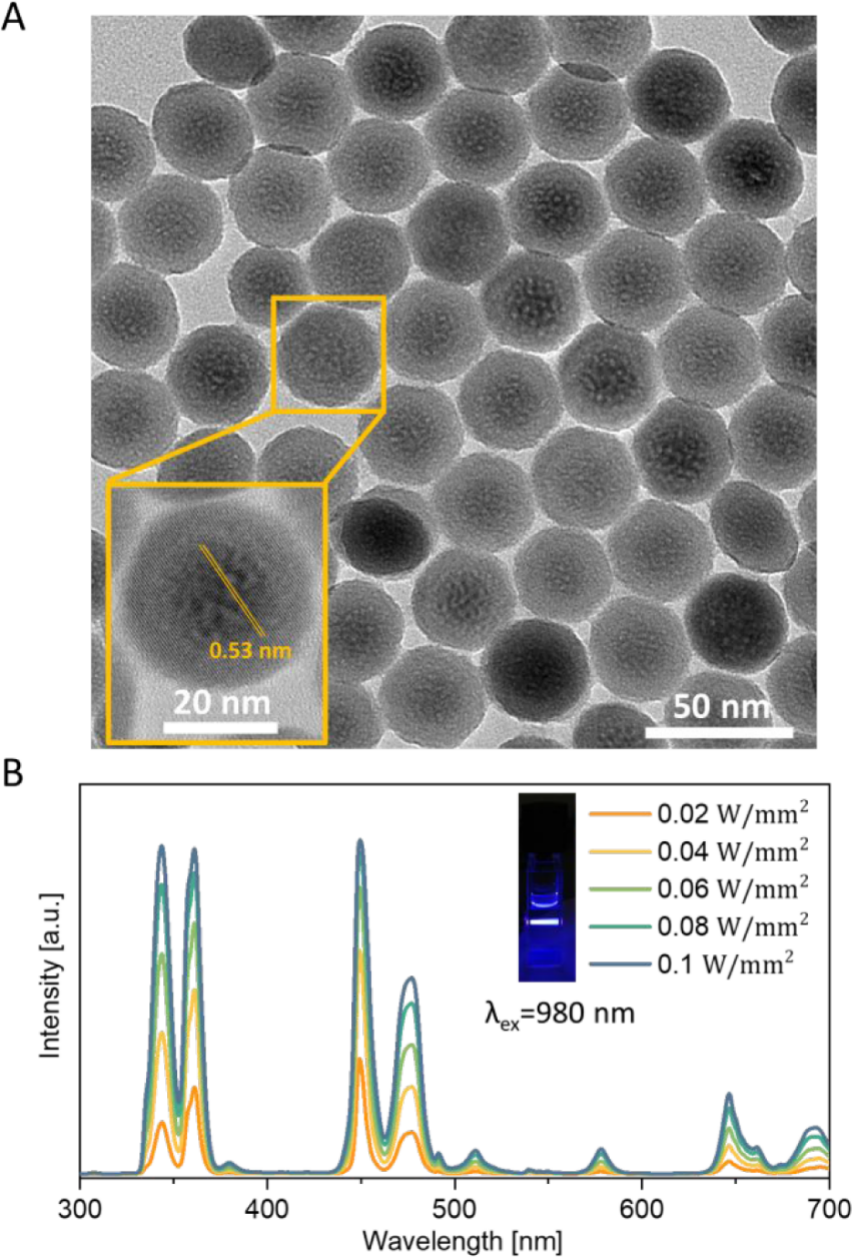
Characterization of NaYF_4_-based UCNPs. (A) Transmission electron microscopy of NaYF_4_-based UCNPs; (B) Emission spectra of NaYF_4_-based UCNPs with different power of the 980 nm laser irradiation (from 0.02 to 0.1 W/mm^2^).

In order to activate motor rotation using UCNPs, the following criteria must be met: (i) Good spectral overlap between the molecular motors’ absorption and UCNPs’ emission; (ii) High photoisomerization quantum yield (**Φ**_**P**_); and (iii) An appropriate rotational speed that enables tracking of the generated metastable isomers. The highly photoefficient molecular motor **1**, which we recently developed,^53^ was chosen as the model system for this proof of concept (Figure 3A, Table 1, and Figure S3). This motor is ideally suited as it features an excitation wavelength around 365 nm (Figure S4) and a long half-life of its metastable *cis*-isomer (Figure 3A, Step IV, ca. 29 h at 20 °C). A simplified rotary cycle is conceived by irradiation at 20 °C, due to the fast THI step (Figure 3A, Step II, ca. 1.5 s at 20 °C). Thus, in principle, with 980 nm irradiation and in the presence of UCNPs, the motor rotation from *cis*-**1**_**st**_ to *cis*-**1**_**mst**_ can be indirectly achieved (Figure 3B).

**Table 1:** Parameters of the Investigated Motors in This Work

motor	*t*_1/2_ (*Z*_mst_ → *Z*_st_)	QY_365_(*E*_st_ **→ ** *Z*_mst_) (%)	QY_365_(*Z*_mst_ **→ ** *E*_st_) (%)
**1**	28.6 h	80	4
**2**	4.6 min	27.7	17.4
**3**	1 to 2 h	-	-
**4**	-	6	1.5
**5**	-	2	1.1

**Figure 3:**
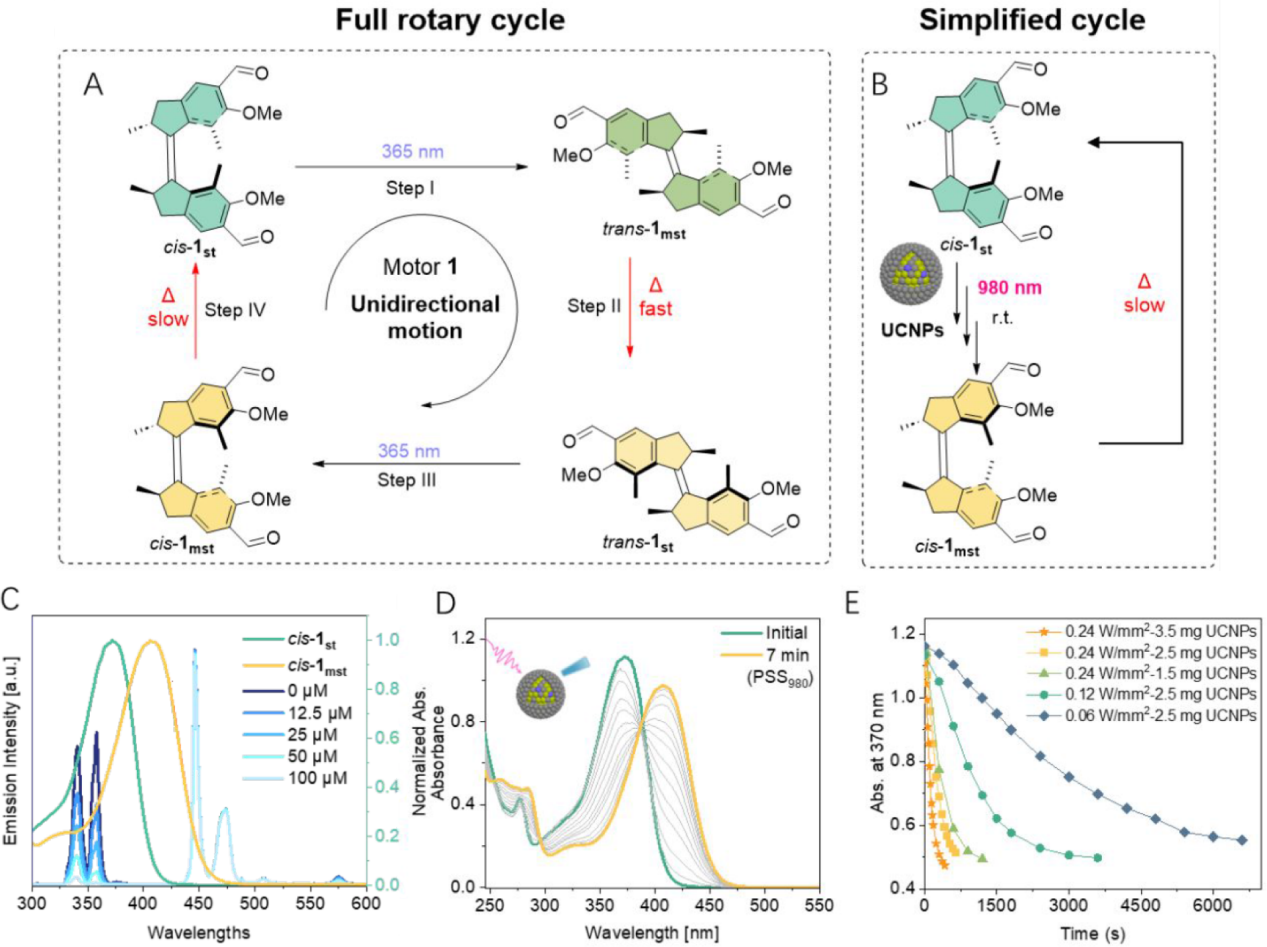
Activation of motor **1** by NIR light at the presence of UCNPs. (A) A full rotary cycle of first-generation motor **1** by 365 nm light. (B) Simplified cycle of activation of first-generation motor **1** by NIR light at the presence of UCNPs at room temperature; (C) Emission spectra of UCNPs after the addition of different concentrations of motor *cis*-**1**_**st**_, overlaid with the normalized absorption spectra of *cis*-**1**_**st**_ and *cis*-**1**_**mst**_. (D) UV–vis spectral change of *cis*-**1**_**st**_ **→** *cis*-**1**_**mst**_ photoisomerization process (50 μM, 0.24 W/mm^2^, 3.5 mg/mL UCNPs, 20 °C, acetonitrile) upon NIR light irradiation. (The intervals are shown in (E)). (E) Comparison of the photoisomerization behavior of *cis*-**1**_**st**_ under different experimental conditions.

## Results and Discussion

Emission quenching experiments were first performed with NaYF_4_ particles to confirm the absorption of upconverted photons by the selected motor candidate. To our delight, the upconversion emission intensity exhibited a clear dependence on the concentration of motor **1**_**st**_. Specifically, two main emission bands of UCNPs, centered at 340 and 365 nm, were significantly quenched upon the addition of motor **1**_**st**_ from 0 to 100 μM (Figure 3C). This result indicates efficient radiative energy transfer from the UCNPs to the molecular motor **1**_**st**_. Upon low-intensity 980 nm irradiation of a solution containing motor *cis*-**1**_**st**_ and a low dosage of UCNPs (50 μM *cis*-**1**_**st**_, 0.12 W/mm^2^, 2.5 mg/mL UCNPs, acetonitrile) at 20 °C, a noticeable color change from colorless to yellowish was observed. This visual change implies successful formation of *cis*-**1**_**mst**_ through a three-step consequential process (*cis*-**1**_**st**_ → *trans*-**1**_**mst**_ → *trans*-**1**_**st**_ → *cis*-**1**_**mst**_) of the motor rotation (Figure 3A). Changes in the UV–vis spectra under 980 nm NIR laser irradiation (Figure S14C) confirm the rotational behavior of motor *cis*-**1**_**st**_, consistent with the behavior observed under direct 365 nm excitation. Remarkably, the photostationary state (PSS) achieved after 1 h of irradiation at 980 nm is comparable to that obtained under direct irradiation at 365 nm (>95%), highlighting the high efficiency of this strategy. Control experiments without NIR light irradiation or UCNPs confirmed the exclusive photochemical process following this strategy (Figures S12 and S13).

Encouraged by the successful demonstration of NIR light-induced motor rotation, we systematically investigated the influence of various parameters, including the NIR light intensity, UCNP concentration, and motor concentration. Increasing the NIR light intensity from 0.06 to 0.24 W/mm^2^ drastically reduced the irradiation time by up to 6-fold to reach the PSS, manifesting the important role of NIR light intensity in this process (Figures 3E and S14). Higher NIR light intensity generates more upconversion photons, accelerating the isomerization of the bulk motor solution. Similarly, reducing the UCNP concentration slightly prolonged the time to reach PSS, while increasing UCNP concentration further shortened the photoisomerization time of the motor solution to reach the PSS (up to 7 min, Figures 3D and S15). The effect of motor concentration on NIR light-powered photoisomerization behavior was also evident, with lower motor concentrations leading to reduced times for the system to reach PSS (Figure S16).

Next, this highly efficient strategy was extended to second-generation molecular motors with different rotary speeds. Motor **2** (Figure 4A) and **3** (Figure 4B) were successfully excited by NIR light, generating metastable isomers with favorable PSS. The core of motor **2** features two five-membered rings at the C=C bond rotary axle, a structure that has been employed in various dynamic systems due to its favorable photochemical properties and suitable rotational speed (at the order of minutes). These included the construction of responsive porous materials,^54–57^ modulation of liquid crystal phases,^58^ and surface^59^ and self-assembled structures.^60^ In the present study motor **2**_**st**_ was selected as a promising candidate due to its high **Φ**_**P**_ (Table 1), attributed to the formylation of the upper half and high PSS at either 365 or 420 nm light irradiation.^61^ An emission quenching experiment was first carried out to confirm the radiative energy transfer mechanism. In this case, the emission intensity of UCNPs was reduced at 340, 360, 450, and 470 nm (Figure 4C), consistent with the wide absorption spectral range of motor **2**_**st**_ from 300 to 480 nm. Due to its short half-life time of the metastable isomer (*t*_1/2_ = 4.6 min at 20 °C), the sample was cooled to 4 °C for *in situ* irradiation to track the photoisomerization process of the motor **2**_**st**_ using UV–vis spectroscopy. Gratifyingly, significant changes in the UV–vis spectra were observed (Figure 4D, gold solid line), which is in line with the formation of the metastable species motor **2**_**mst**_. The subsequent thermal helix inversion (THI) step showed almost complete regeneration of the stable isomer motor **2**_**st**_ (Figure 4D, light green dashed line), revealing a robust photochemical behavior of the motor **2** under NIR light irradiation conditions. The PSS obtained upon 980 nm laser irradiation was lower than that using direct irradiation at 365 nm (Figure 4D, orange dashed line), which can be attributed to the competing photoisomerization process (Figures 4A and S5).

**Figure 4:**
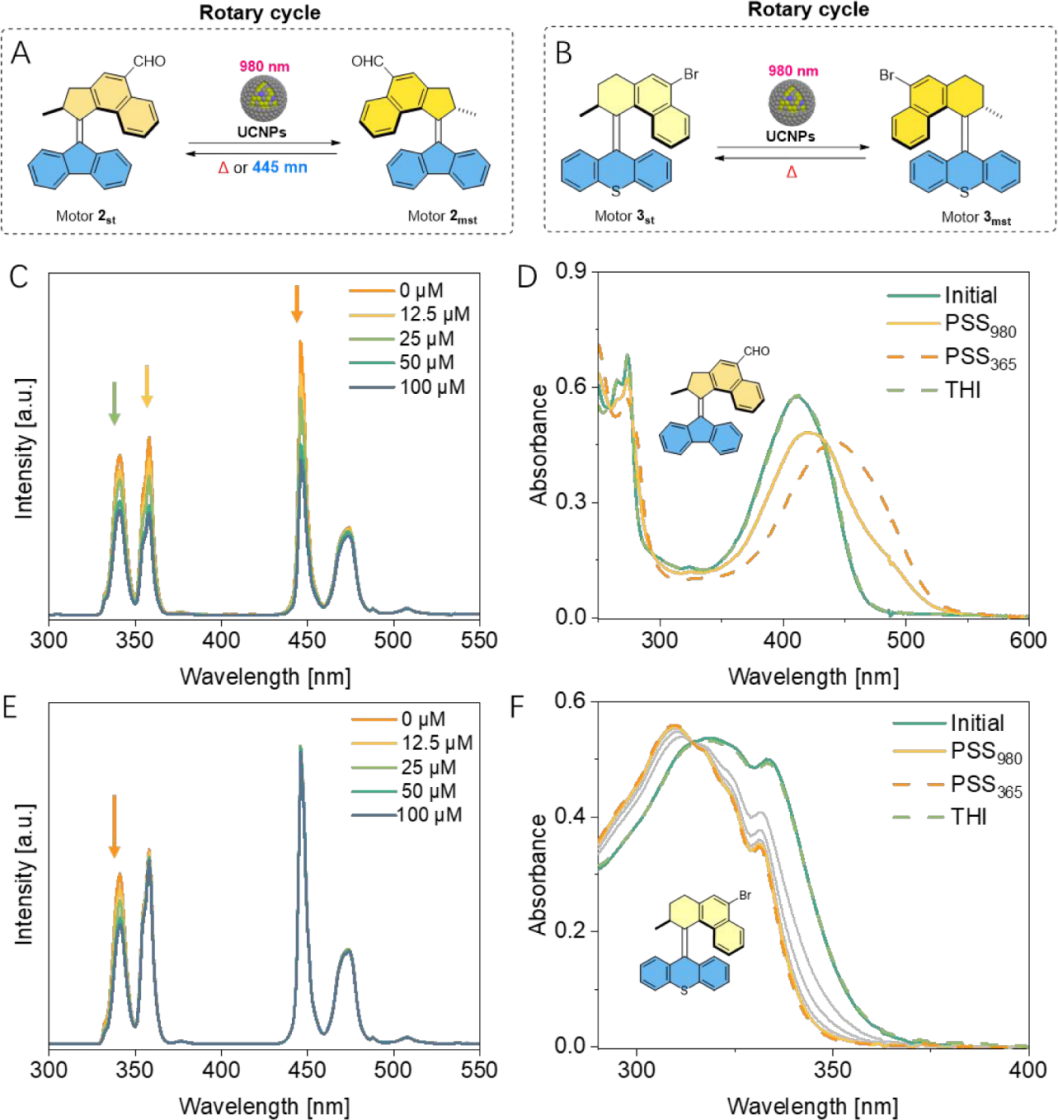
Rotation cycle of Motor **2** (A) and **3** (B) by UCNPs. Emission spectra of UCNPs were obtained using different concentrations of (C) motor **2**_**st**_ or (E) motor **3**_**st**_. Activation of second-generation (D) motor **2**_**st**_ (4 °C) and (F) motor **3**_**st**_ (20 °C) by NIR or 365 nm light was observed in the presence of UCNPs in acetonitrile.

Similar phenomena were observed when bistable overcrowded alkene-based photoswitch^17^ **4** or **5** (Figures S6, S7, and S13) were tested. These attractive photoswitches have been applied in material science^54,62^ and dynamic systems,^63,64^ and allow to prove the interference of the competing photo back-isomerization process as a result of the visible light region emission by UCNPs due to the absorption of metastable isomers (Figures S7 and S8). These results indicated the importance of **Φ**_**P**_ for competing photochemical processes (Table 1). It should be emphasized that the emission at 450–500 nm in the motor **1** system did not affect the PSS of metastable species due to high photoselectivity in the *cis*-**1**_**st**_ → *cis*-**1**_**mst**_ process and low **Φ**_**P**_ for competing photochemical process (Table 1). Next, this strategy was further extended to another widely applicable second-generation motor scaffold **3**, which has a half-life of approximately 1 h (Figure 4B) and shows great potential for use in dynamic assembly systems^65,66^ and biological applications.^67^ Due to the negative photochromism of this core and the absence of overlapping absorption bands at wavelengths longer than 370 nm with the emission spectrum of UCNPs (Figure S6), motor **3**_**st**_, in principle, should show comparable PSS when exposed to NIR light, relative to direct excitation at 365 nm. Emission quenching experiments confirmed that the upconversion emission centered at 340 and 365 nm was absorbed, with no detectable reduction in the UCNPs emission spectrum at wavelengths longer than 360 nm (Figure 4E). Indeed, both NIR and 365 nm light irradiation induced similar photoisomerization behavior (Figure 4F, gold solid line for 980 nm irradiation and orange dashed line for 365 nm light irradiation). The subsequent THI step fully regenerated the stable isomer **3**_**st**_ (Figure 4F, light green dashed line). This approach successfully demonstrates the versatile NIR light activation of second-generation molecular motors with high stability and efficiency, representing a significant advantage over our previously reported 2PA methodology.^41,68^

## Conclusion

In summary, we developed a general, simple, and practical strategy to fuel light-driven molecular motors by NIR light. The UCNPs serve as light nanotransducers, generating UV and visible photons from NIR light, which are absorbed by molecular motors to trigger unidirectional rotary motion. This radiative energy transfer mechanism demonstrates a performance comparable to direct UV-light excitation. A unique and highly efficient approach has been established to replace harmful UV irradiation for powering molecular motors, offering significant advantages in biological systems and other environments that are sensitive to UV light or require deeper light penetration, such as bulk solid materials. In addition, no complicated prefunctionalization of molecules is required in this doped system. Our approach exhibits generality and scope in activating molecular motors with NIR light, presenting a significant step forward toward real applications of molecular machines.

## Supplementary Material





## Notes

Details on the synthesis and structural characterizations of UCNPs and compounds, NMR spectra, additional experimental details, materials, and methods, including Figures S1–S20 (PDF)
(PDF)
