# Supplementary material for: Highly Efficient Near-Infrared Light-Driven Molecular Motor Rotation Enabled by Upconversion Nanoparticles as Nanoscale Light Sources
Source: J Am Chem Soc. 2025 Jul 17;147(30):26797–803. doi: 10.1021/jacs.5c07953 (PMC12314898; doi:10.1021/jacs.5c07953)
Supplement: Supplementary file 1 [file ja5c07953_si_001.pdf]

Highly Efficient Near-Infrared Light-Driven Molecular Motor Rotation Enabled by Upconversion Nanoparticles as Nanoscale Light Sources

Jinyu Sheng, [‡, †, ∇, *] Youxin Fu, [‡, #, ∇] Kefan Wu, [ƻ, ∇] Thomas Freese,[‡] Hong Zhang,[ƻ, *] and Ben L. Feringa[‡, *]

[‡]*Stratingh Institute for Chemistry, University of Groningen, Nijenborgh 3, 9747 AG Groningen, The Netherlands.*

[†]*College of Chemistry, Chemical Engineering and Materials Science, Soochow University, Suzhou, Jiangsu 215123, China.*

[#] *College of Science, Nanjing Forestry University, Nanjing, 210037, P.R. China.*

[ƻ] *Van 't Hoff Institute for Molecular Sciences, University of Amsterdam, Science Park 904, 1098 XH Amsterdam, The Netherlands.*

[∇]These authors contributed equally.

Contents

1. General Information 2

2. Synthesis and Characterizations of UCNPs and motors 4

Synthesis of 25 nm $NaYF_4$:75%Yb^{3+},0.5%Tm^{3+} bare cores 4

Synthesis of 35 nm $NaYF_4$:75%Yb^{3+},0.5%Tm^{3+} @$NaYF_4$ core-shell nanoparticle 4

Synthesis of molecular motors 4

3. Characterization of UCNPs by TEM and DLS. 7

4. Unidirectional study and fatigue study of motor 1 by ^{1}H NMR. 8

5. Overlap of the emission spectrum of UCNP and Absorption spectra of motors 9

6. Control UV-Vis experiments 13

7. UV-Vis studies of NIR light powered photoisomerization of molecular motors. 14

8. Local heating effect inverstigation induced by NIR absorption. 18

9 NMR data 19

10. References 23

1. General Information

All reagents were obtained from commercial sources such as Aldrich, TCI, Fluorochem, combi-Blocks and were used as received. Anhydrous DCM, THF, Diethyl ether was obtained from a solvent purification system (MBRAUN SPS systems, MBSPS-800). Flash column chromatography was performed using silica gel (SiO_2) purchased from Merck (type 9385, 230-400 mesh) or using a Büchi Reveleris purification system with Büchi cartridges. NMR spectra were recorded on Varian AMX400 (^{1}H: 400 MHz, ^{13}C: 101 MHz) and Varian Unity Plus (^{1}H: 500 MHz, ^{13}C: 126 MHz) spectrometers. Chemical shifts are quoted in parts per million (ppm) relative to the residual solvent signal (for $CDCl_3$ δ 7.26 for ^{1}H, δ 77.16 for ^{13}C and for CD_2Cl_2 δ 5.32 for ^{1}H, δ 53.84 for ^{13}C). For ^{1}H NMR spectroscopy, the splitting pattern of peaks is designated as follows: s (singlet), d (doublet), t (triplet), m (multiplet), br (broad), or dd (doublet of doublets). High resolution mass spectrometry (ESI or APCI-MS) was performed on a LTQ Orbitrap XL spectrometer with ESI ionization. The UV/Vis, irradiation experiments were performed using fiber-coupled LEDs (F365F1, M455F1) obtained from Thorlabs Inc or 980 nm laser. UV/Vis absorption spectra were measured on a Hewlett-Packard 8453 diode array spectrometer in a 1 cm quartz cuvette. Inc.

The laser source used in our study is a CW 980 nm diode laser, coupled through a collimation lens to produce a near-parallel beam. The beam was directed onto the sample at normal incidence through a 1 cm quartz cuvette. The beam spot size at the sample was experimentally measured to be approximately 0.5 mm in diameter. the normalized profile shows a clear peak with a FWHM of 2 scanning units, confirming strong intensity concentration at the beam center 974 nm (Figure S1A). The optical path was kept short (~2 cm) and enclosed to minimize beam divergence and ambient scattering. No focusing optics were used beyond the initial collimator to ensure a uniform excitation area across the sample volume.

Reagents for UCNP synthesis. $LnCl_3•6H_2O$ (Ln: Er, Yb, Y, 99.9% trace metals basis), Ln_2O_3 (Ln: Y, Tm, Yb, Nd, 99.9% trace metals basis), oleic acid (OA), 1-octadecene (ODE, 90%, technical grade), sodium hydroxide (NaOH, 97%), trifluoroacetic acid (TFA, 99%), sodium trifluoroacetate (Na-TFA, 98%), *N, N*-dimethylformamide (DMF 99%), and Nitrosonium tetrafluoroborate ($NOBF_4$ 95%) were obtained from Sigma Aldrich. Acetone (>99%), ethanol (96%), and cyclohexane were purchased from VWR chemicals. Ammonium fluoride (NH_4F, 98%+ extra pure) was purchased from Acros organics.

A PHILIPS CM 120 Cryo electron microscope with 120 keV was used acquire the Transmission Electron Microscopy (TEM) images of **UCNP**s. All the motors were synthesized by our reported methods and were fully characterized.

Sample preparation (with UCNPs addition). Stock solution of **Motors** (in MeCN), and **UCNP**s (15 mg/mL, in MeCN) were prepared. A solution of **Motor/UCNPs** with different concentrations was prepared in MeCN (2mL) in a quartz cuvette without a degassing procedure needed. Near infrared continuous wave laser (MDL-H-980nm, MDL-N-800nm, order from Changchun New Industries Optoelectronics Tech. Co.) was used as light source for motor rotation and positioned at a fixed distance to the cuvette; the progress of photoreaction was monitored by UV-Vis absorption spectra.

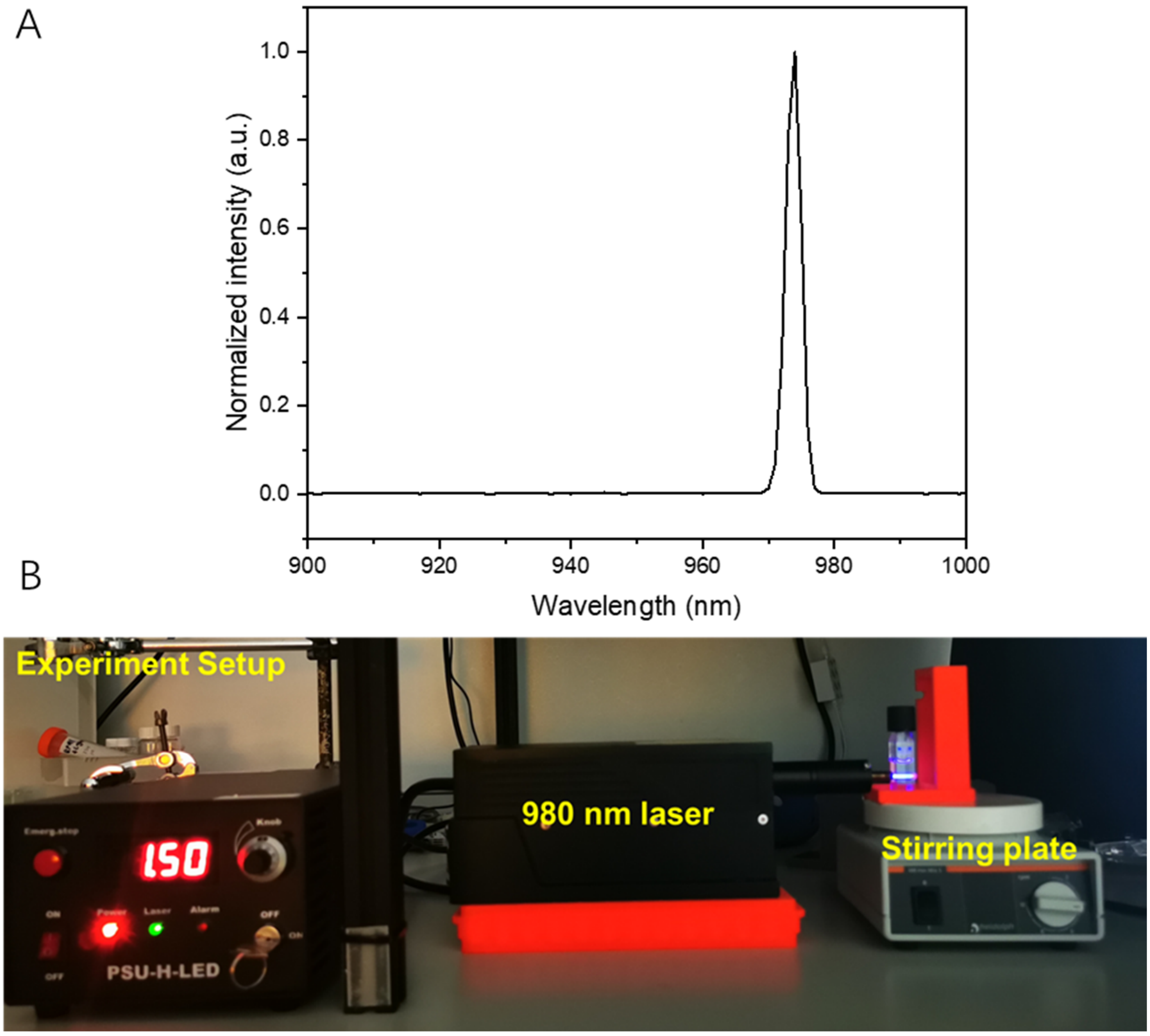

Figure. S1. A. The beam profile of 980 nm CW laser. B. Experimental set-up for the NIR light-induced unidirectional motor rotation.

2. Synthesis and Characterizations of UCNPs and motors

Synthesis of 25 nm $NaYF_4$:75%Yb^{3+},0.5%Tm^{3+} bare cores

The cores $NaYF_4$:75%Yb^{3+},0.5%Tm^{3+} were synthesized by a well-developed thermal decomposition method[1]. First, 0.75 mmol $YbCl_3·6H_2O$, 0.005 mmol $TmCl_3·6H_2O$, and 0.245 mmol $YCl_3·6H_2O$ were added to a mixture of 6 mL oleic acid (OA, 90%) and 15 mL 1-octadecene (ODE, 90%) in a 100 mL three-necked round-bottom flask. Next, the chlorides were dissolved at 160°C for 30 min under a nitrogen flow. After the solution was cooled down to room temperature, 2.5 mmol NaOA and 4 mmol NH_4F were added. The mixture was stirred and heated to 100°C for 1 h in vacuum until the powder dissolved. Subsequently, the temperature of the resulting solution was quickly increased to 300 °C and maintained for 90 min in a nitrogen atmosphere. The mixture was cooled down to room temperature, and the products were collected by centrifugation at 8000 rpm for 10 min, washed with ethanol twice, and finally dispersed in 4 mL of cyclohexane.

Synthesis of 35 nm $NaYF_4$:75%Yb^{3+},0.5%Tm^{3+} @$NaYF_4$ core-shell nanoparticle

Core-shell nanoparticle following a well-developed method.[2] Typically, 1 mmol $Y(CF_3COO)_3·3H_2O$ and 1 mmol CF_3COONa were mixed with 3 mL oleic acid and 7.5 mL 1-octadecene in a 100 mL flask. The mixture was heated to 150 °C to form a homogenous shell precursor solution and then cooled down to room temperature. Subsequently, 1 mL of the prepared bare core in cyclohexane ($NaYF_4$:75%Yb^{3+},0.5%Tm^{3+}, 0.25 mmol) was added into a 100 mL three-neck round-bottom flask followed by 3 mL OA and 7.5 mL ODE addition. The mixture was then heated up to 100 °C in vacuum for 30 min to remove cyclohexane and residual air. Next, the solution was heated up to 300 °C under a nitrogen atmosphere and stirred for 5 min. The as-obtained $NaYF_4$ shell precursors (10.5 mL) were injected into the solution in four 15-min intervals. After the injection of all shell precursors, the mixture was allowed to ripen for 45 min before cooling down. The core-shell UCNPs ($NaYF_4$:75%Yb^{3+},0.5%Tm^{3+}@$NaYF_4$) were collected by centrifugation with ethanol twice and redispersed in 5 mL cyclohexane.

To facilitate the transfer of UCNPs from a hydrophobic medium (e.g., cyclohexane) to a hydrophilic medium (e.g., MeCN), a ligand removal strategy is often employed due to its versatility. Here, 2 mL of the as-synthesized UCNP and 2 mL of DMF were combined in a 10 mL vial, followed by the addition of 10 mg of Nitrosonium tetrafluoroborate ($NOBF_4$). The resulting mixture was sonicated for 20 min and then centrifuged at 10000 rpm for 10 min to precipitate the quasi-ligand- free UCNPs in DMF. The UCNPs can then be easily redispersed in 2 mL of MeCN.

Synthesis of molecular motors

Motor **1** was synthesized according to a reported procedure.[3] Analytical data are in agreement with the literature.

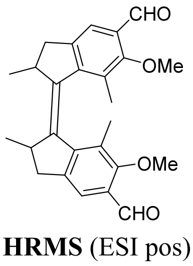

Note: The motor solution should be covered with Aluminum foil to avoid isomerization by ambient light.

^{1}H NMR (400 MHz, $CDCl_3$) δ 10.37 (s, 2H), 7.63 (s, 2H), 3.85 (s, 7H), 3.43 (p, *J* = 6.9 Hz, 2H), 3.23 (dd, *J* = 14.9, 6.2 Hz, 2H), 2.55 (d, *J* = 14.8 Hz, 2H), 1.51 (s, 7H), 1.09 (d, *J* = 6.7 Hz, 7H).
^{13}C NMR (101 MHz, $CDCl_3$) δ 190.4, 161.3, 149.1, 144.0, 142.2, 129.8, 128.0, 121.7, 62.8, 43.0, 39.9, 20.0, 14.5.
HRMS (ESI pos) calcd $C_{26}H_{29}O_4$ $[M+H]^+$: 405.2060, found 405.2058.

1-(9H-fluoren-9-ylidene)-2-methyl-2,3-dihydro-1H-cyclopenta[a]naphthalene-5-carbaldehyde.

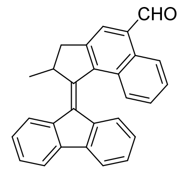

Under a N_2 atmosphere, 9-(5-bromo-2-methyl-2,3-dihydro-1H-cyclopenta[a]naphthalen-1-ylidene)-9H-fluorene (800.00 mg, 1.89 mmol) was dissolved in THF (20 mL) and the mixture was cooled to -78 °C. N-butyllithium (1.6M in hexane, 1.42 mL, 2.27 mmol) was slowly added to the solution which was stirred for 15 min. Then anhydrous DMF (2.5 mL) was added and the mixture was stirred for 1 h at room temperature. The reaction was quenched by saturated aqueous NH_4Cl, and the mixture was extracted with EtOAc, washed with water, brine, dried over Na_2SO_4, filtrated and concentrated in vacuo. The crude product was purified by column chromatography (SiO_2, pentane: EtOAc = 1:1) to afford motor **2** as a yellow solid (530.0 mg, 1.37 mmol, 72%). Analytical data in agreement with the literature.[4]

^{1}H NMR (500 MHz, CD_2Cl_2) δ 10.50 (s, 1H), 9.27 (d, *J* = 8.6 Hz, 1H), 8.11 (s, 1H), 8.02 – 7.98 (m, 1H), 7.93 (d, *J* = 8.5 Hz, 1H), 7.86 – 7.81 (m, 1H), 7.74 (d, *J* = 7.5 Hz, 1H), 7.66 (t, *J* = 7.7 Hz, 1H), 7.44 – 7.37 (m, 3H), 7.22 (t, *J* = 7.5 Hz, 1H), 6.75 (t, *J* = 7.6 Hz, 1H), 6.63 (d, *J* = 7.9 Hz, 1H), 4.42 – 4.35 (m, 1H), 3.65 (dd, *J* = 15.0, 5.7 Hz, 1H), 2.88 (d, *J* = 15.1 Hz, 1H), 1.40 (d, *J* = 6.8 Hz, 3H).

^{13}C NMR (101 MHz, $CDCl_3$) δ 193.07, 149.06, 145.31, 143.60, 140.73, 140.21, 139.58, 136.87, 133.94, 133.37, 132.54, 130.32, 130.25, 128.44, 128.06, 128.02, 127.94, 127.44, 127.38, 126.33, 126.10, 125.45, 124.64, 120.00, 119.32, 45.56, 41.63, 19.24.

HRMS (APCI pos) calcd $C_{28}H_{21}O$ $[M+H]^+$: 373.1587, found 373.1588.

9-(9-bromo-3-methyl-2,3-dihydrophenanthren-4(1H)-ylidene)-9H-thioxanthene.

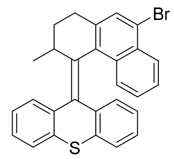

Lawesson's reagent (3.5 g, 8.4 mmol) and 9H-thioxanthen-9-one (848.0 mg, 4 mmol) were dissolved in dry toluene (15 mL) in a vial under N_2 atmosphere. The mixture was heated at 90 °C for 1 h. Then the mixture was poured onto the column to purify by quick column chromatography (SiO_2, pentane:CH_2Cl_2 = 3:1). The thioketone fraction was collected and concentrated under reduced pressure to yield the crude thioketone as a dark brown solid. In another two-neck flask under N_2 atmosphere, hydrazone (151.0 mg, 0.5 mmol) was dissolved in DMF (10 mL). The mixture was cooled to -40 °C, and bis(trifluoroacethoxy)iodobenzene (236.0 mg, 0.55 mmol) in DMF (3 mL) was then added to the stirred mixture. The mixture was stirred for 3 min while the color turned to pink, indicative of the *in situ* formation of the diazo compound. A solution of the 9H-thioxanthene-9-thione (114.0 mg, 0.5 mmol) in dry DMF (5 mL) was added. The mixture was allowed to warm to room temperature and stirred for 24 h. The mixture was poured into water and extracted with EtOAc. Then the crude was concentrated and purified by column chromatography (SiO_2, pentane) to afford white episulfide. Next the episulfide was dissolved in toluene, HMPT (Tris(dimethylamino)phosphine) (0.4 mL) was added and the mixture was stirred at 65 °C for another 16 h. The mixture was extracted with EtOAc, washed with water, brine, dried over Na_2SO_4, filtrated and concentrated in vacuo. The crude product was purified by column chromatography (SiO_2, pentane) to afford motor **3** as a white solid (62.4 mg, 0.13 mmol, 25% two steps). Analytical data in agreement with the literature.[4]

^{1}H NMR (400 MHz, CD_2Cl_2) δ 8.01 (d, *J* = 8.5 Hz, 1H), 7.74 (s, 1H), 7.64 (td, *J* = 7.5, 1.3 Hz, 2H), 7.54 (d, *J* = 8.5 Hz, 1H), 7.41 – 7.33 (m, 2H), 7.32 – 7.20 (m, 2H), 7.03 (t, *J* = 7.7 Hz, 1H), 6.77 (td, *J* = 7.6, 1.4 Hz, 1H), 6.42 (td, *J* = 7.5, 1.2 Hz, 1H), 6.32 (d, *J* = 7.7 Hz, 1H), 3.90 (td, *J* = 10.2, 5.2 Hz, 1H), 3.07 (ddd, *J* = 15.1, 10.5, 7.7 Hz, 1H), 2.95 (ddd, *J* = 15.1, 6.5, 2.9 Hz, 1H), 2.67 – 2.55 (m, 1H), 1.48 (dd, *J* = 19.8, 3.5 Hz, 1H), 0.61 (d, *J* = 6.8 Hz, 3H).

^{13}C NMR (101 MHz, $CDCl_3$) δ 139.4, 138.7, 138.2, 136.6, 136.1, 134.6, 134.2, 132.4, 131.4, 130.5, 129.8, 128.5, 128.4, 128.0, 126.9, 126.7 (2*CH), 126.3, 126.1, 126.1, 125.8, 125.7, 125.6, 122.0, 31.0, 30.8, 28.8, 21.8.

HRMS (APCI pos) calcd $C_{28}H_{22}BrS$ $[M+H]^+$: 469.0620, found 469.0621.

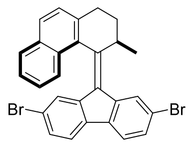

4-(2,7-Dibromo-9*H*-fluoren-9-ylidene)-3-methyl-1,2,3,4-tetrahydrophenanthrene. Motor **4** was prepared by following the procedure previously reported. Analytical data in agreement with the literature.[5]

^{1}H NMR (400 MHz, $CDCl_3$) δ 8.19 (d, *J* = 1.7 Hz, 1H), 7.92 (dd, *J* = 12.3, 8.1 Hz, 2H), 7.79 (d, *J* = 8.5 Hz, 1H), 7.63 (d, *J* = 8.1 Hz, 1H), 7.54 (dd, *J* = 8.1, 1.6 Hz, 1H), 7.50 - 7.37 (m, 3H), 7.26 - 7.21 (m, 1H), 7.17 (dd, *J* = 8.1, 1.8 Hz, 1H), 5.96 (d, *J* = 1.7 Hz, 1H), 4.20 (p, *J* = 7.2 Hz, 1H), 2.86 - 2.75 (m, 1H), 2.64 - 2.43 (m, 2H), 1.30 (d, *J* = 6.9 Hz, 3H), 1.25 - 1.17 (m, 1H).

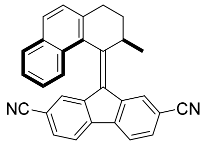

9-(3-Methyl-2,3-dihydrophenanthren-4(1*H*)-ylidene)-9H-fluorene-2,7-dicarbonitrile. Under a N_2 atmosphere, compound **4** (77.0 mg, 150 µmol), $Zn(CN)_2$ (21.0 mg, 180 µmol), *t*-BuXPhos-Pd-G3 (12 mg, 15.0 µmol) and *t*-BuXPhos (17.2 mg, 40.0 µmol) were dissolved in degassed DMF/H_2O mixture (10 mL of DMF, 0.1 mL of H_2O) and the mixture was heated at 100 °C for 3 h. Subsequently, the mixture was poured into water (100 mL), and extracted by ethyl acetate (3x10 mL). The organic layer was washed with water, brine, dried over Na_2SO_4, filtered, and concentrated *in vacuo*. The crude product was purified by column chromatography (SiO_2, pentane: ethyl acetate = 2:1) to afford **5** as pale yellow solid motor **5** (55.0 mg, 135 µmol, 90%). Analytical data in agreement with the literature.[5]

^{1}H NMR (400 MHz, $CDCl_3$) δ 8.40 (d, *J* = 1.3 Hz, 1H), 7.96 (ddd, *J* = 12.7, 11.2, 8.4 Hz, 3H), 7.81 – 7.73 (m, 2H), 7.69 (d, *J* = 8.5 Hz, 1H), 7.51 (d, *J* = 8.3 Hz, 1H), 7.45 – 7.37 (m, 2H), 7.22 (ddd, *J* = 8.3, 6.8, 1.3 Hz, 1H), 6.19 (dd, *J* = 1.3, 0.7 Hz, 1H), 4.23 (q, *J* = 7.1 Hz, 1H), 2.86 (dt, *J* = 14.4, 3.7 Hz, 1H), 2.65 – 2.47 (m, 2H), 1.34 (d, *J* = 7.0 Hz, 3H), 1.30 – 1.25 (m, 1H).

^{13}C NMR (101 MHz, $CDCl_3$) δ 151.5, 142.5, 141.1, 140.7, 138.8, 138.7, 132.5, 131.7, 131.5, 131.2, 130.7, 130.5, 130.3, 129.1, 129.0, 128.8, 127.6, 126.1, 125.7, 124.0, 121.3, 120.5, 119.7, 119.0, 111.9, 111.2, 35.7, 30.3, 29.6, 20.9.

HRMS (ESI pos) calcd $C_{30}H_{20}N_2$ $[M]^+$: 408.1621, found 408.1617.

3. Characterization of UCNPs by TEM and DLS.

A PHILIPS CM 120 Cryo electron microscope with 120 keV was used to take the TEM images. The sample in cyclohexane (15 mg/mL) was diluted by 12.5× (UCNP 1). The sample grid was prepared by dropping 5 µL of the solution onto an ultrathin carbon film coated copper grid. After 30 sec of drying the grid was washed with 5 µL ethanol (EtOH) and any surplus solvent was dried on a filter paper.

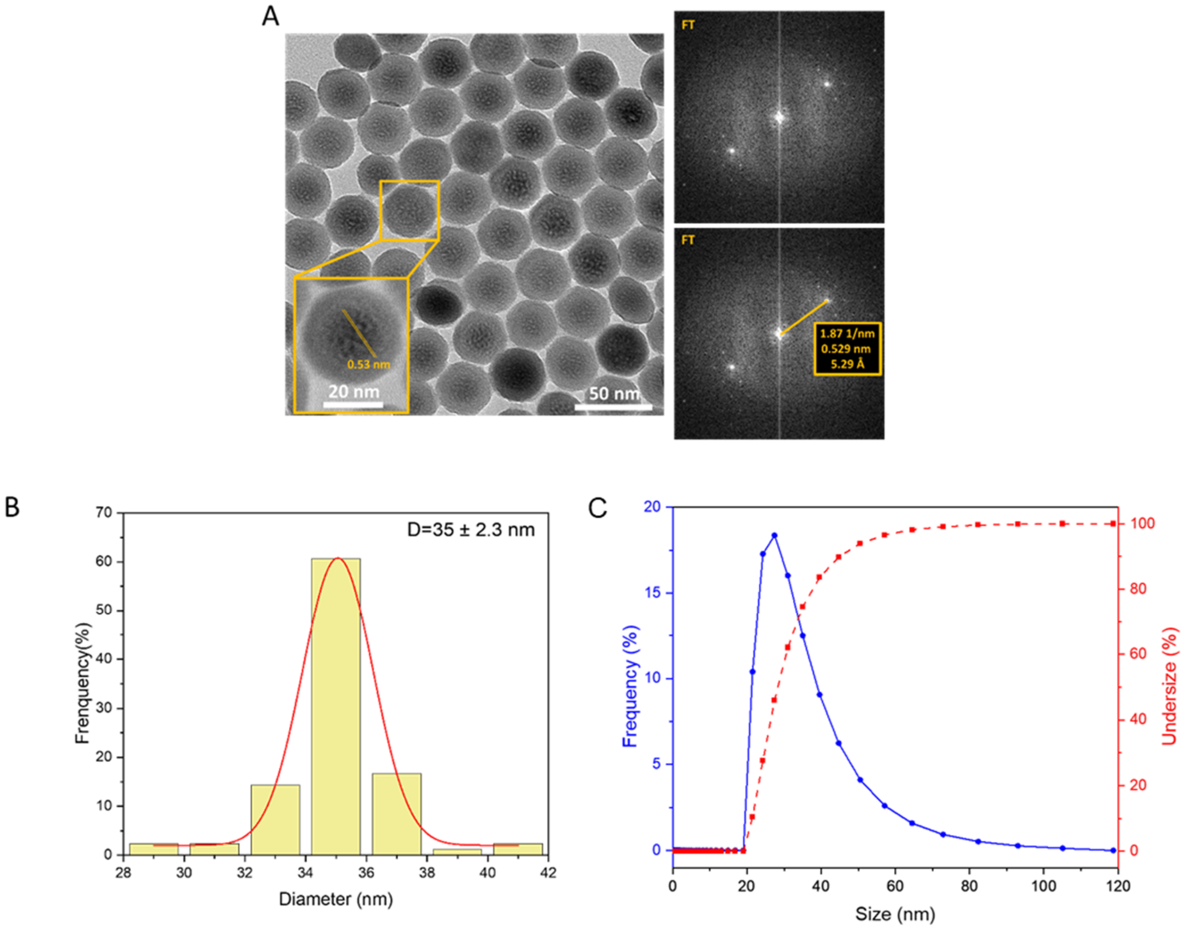

Figure S2. Characterization of UCNPs. A. Transmission electron microscopy of **UCNP** (15 mg/mL diluted in cyclohexane), at a magnification of *100000; inset: zoom on nanoparticle with a magnification of *160000 (left). Fourier transform of the zoomed nanoparticle and atom distance (right). B. The size distribution histograms of **UCNP**s, the size distribution histograms were obtained from the statistical result of more than 50 particles. C. The DLS analysis of UCNPs dispersed in MeCN after ligand removal using $NOBF_4$.

4. Unidirectional study and fatigue study of motor 1 by ¹H NMR.

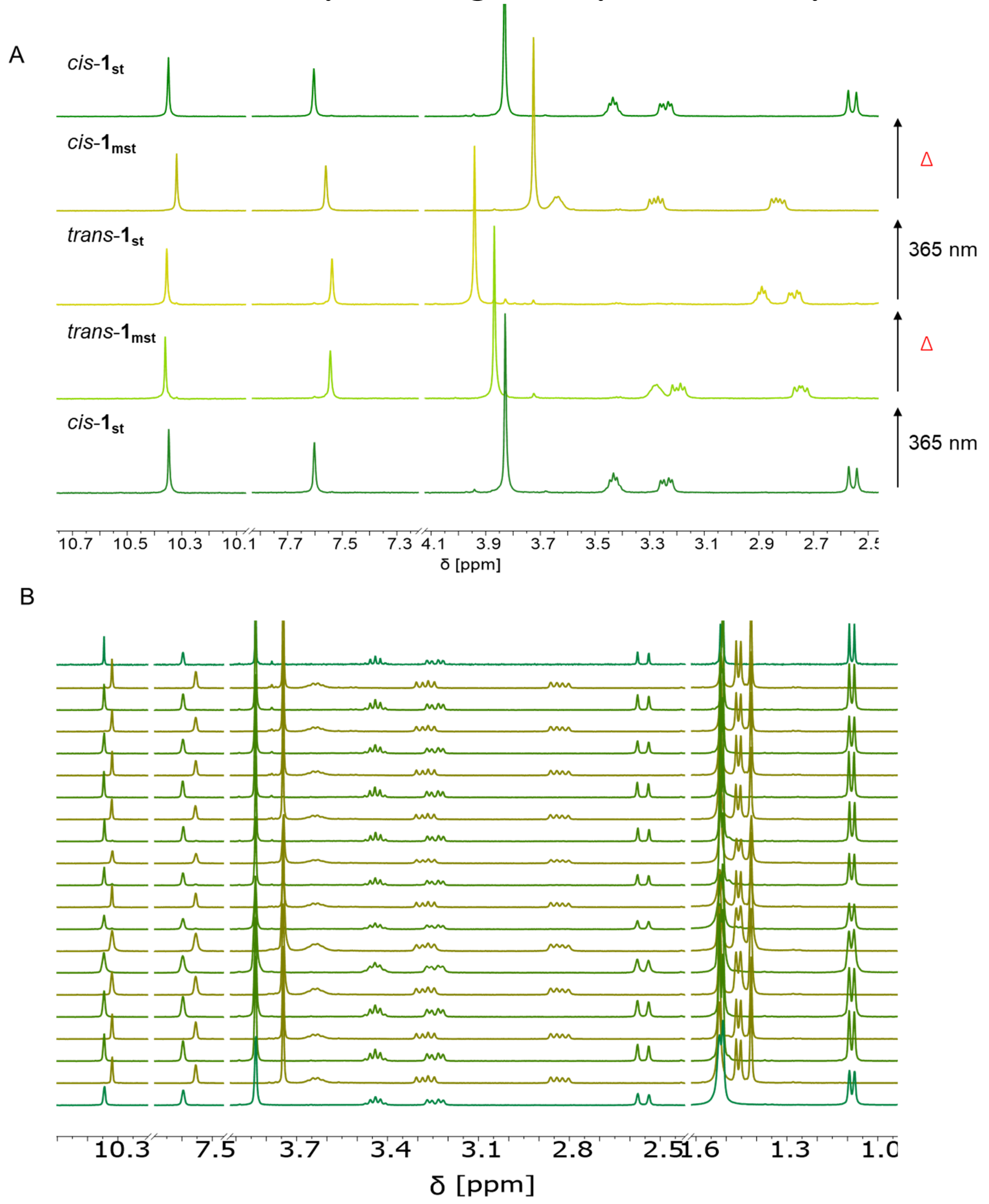

Figure S3. A. Rotary cycle of Motor **1** monitored by ¹H NMR spectroscopy. The spectra were measured and recorded at -55 °C. B. Fatigue studies of Motor **1** by ¹H NMR spectroscopy between *cis*-**1**$_{\mathbf{mst}}$ and *cis*-**1**$_{\mathbf{st}}$. The *cis*-**1**$_{\mathbf{mst}}$ sample was generated from irradiation of the *cis*-**1**$_{\mathbf{st}}$ sample, and the *cis*-**1**$_{\mathbf{st}}$ was recovered by heating the sample to 40 °C. These procedures were repeated for 10 times.

5. Overlap of the emission spectrum of UCNP and Absorption spectra of motors

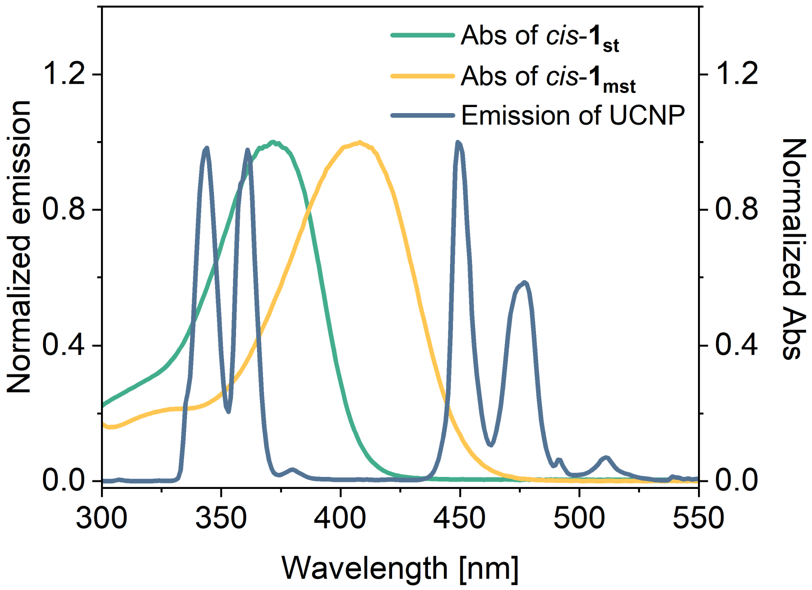

Figure S4. Overlap of the emission spectrum of UCNP and absorption spectra of motor **M1**. The normalized *cis*-$\mathbf{1}_{mst}$ is generated from the PSS_{365} of an irradiated *cis*-$\mathbf{1}_{st}$ sample.

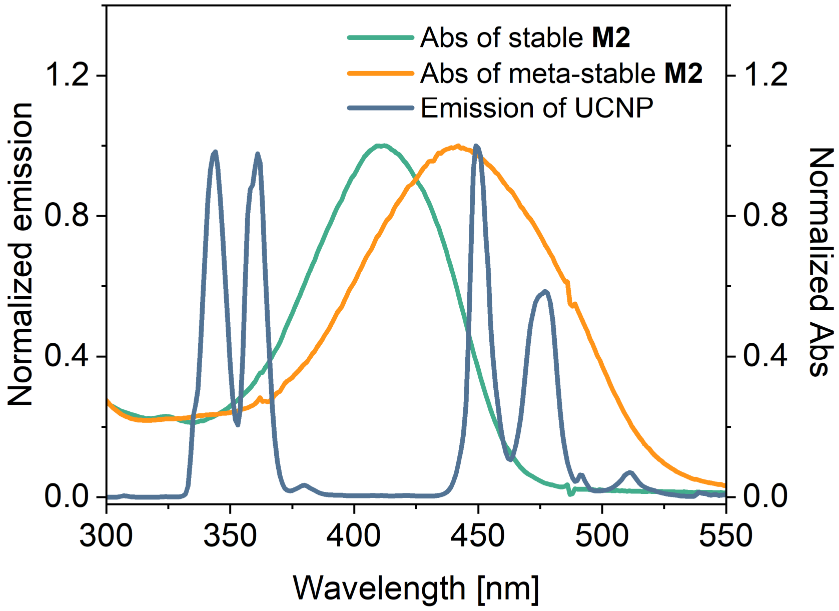

Figure S5. Overlap of the emission spectrum of UCNP and absorption spectra of motor $\mathbf{M2}_{st}$. The normalized $\mathbf{M2}_{mst}$ is generated from the PSS_{365} of an irradiated stable $\mathbf{M2}_{st}$ sample.

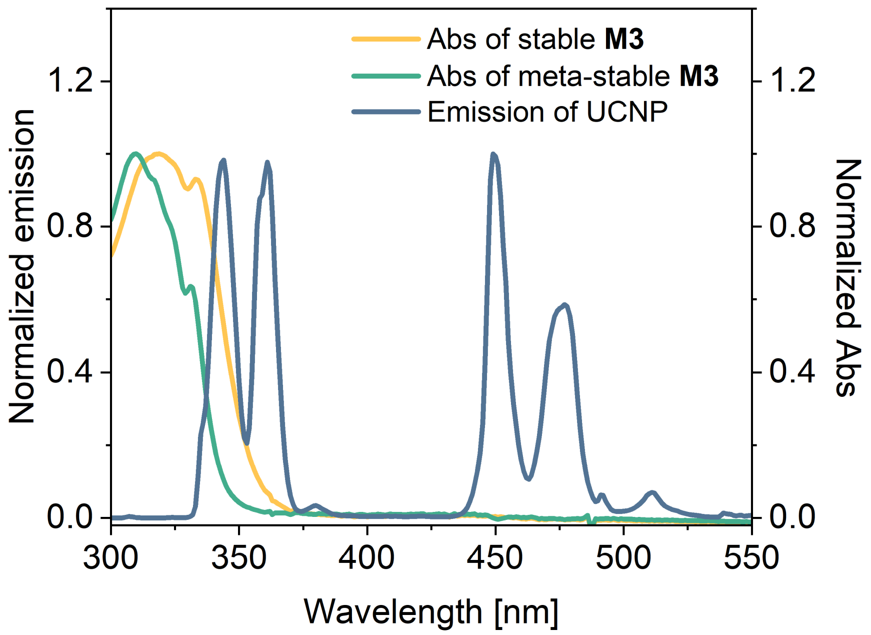

Figure S6. Overlap of the emission spectrum of UCNP and absorption spectra of motor **M3**$_{\mathbf{st}}$. The normalized **M3**$_{\mathbf{mst}}$ is generated from the PSS$_{365}$ of an irradiated **M3**$_{\mathbf{st}}$ sample.

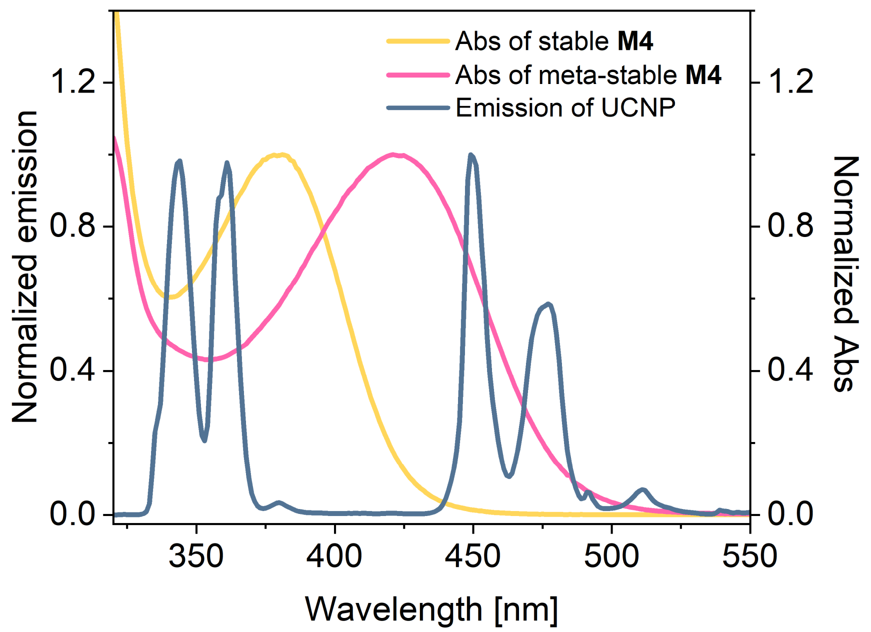

Figure S7. Overlap of the emission spectrum of UCNP and absorption spectra of photoswitch **M4**. The normalized **M4**$_{\mathbf{mst}}$ is generated from the PSS$_{365}$ of an irradiated stable **M4**$_{\mathbf{st}}$ sample.

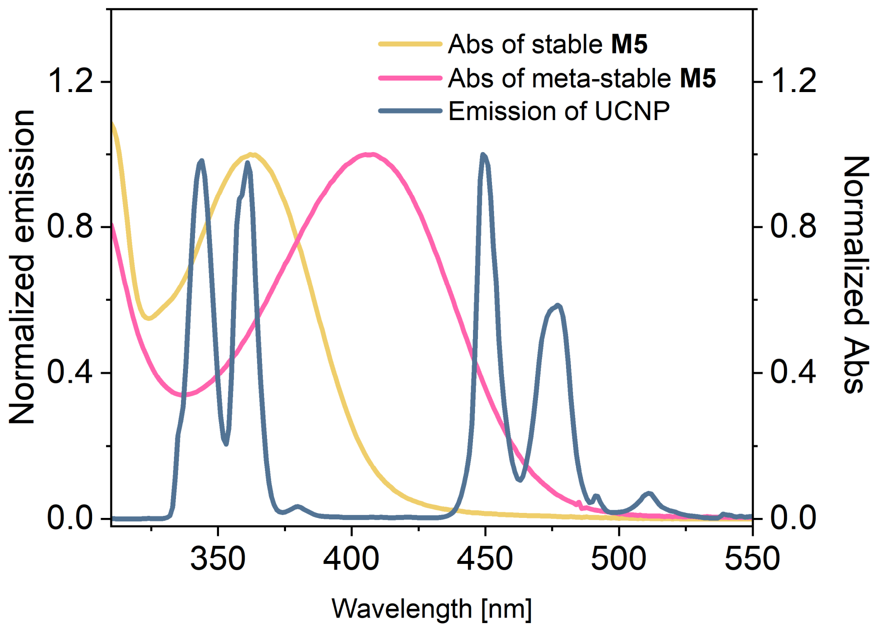

Figure S8. Overlap of the emission spectrum of UCNP and absorption spectra of photoswitch **M5**. The normalized **M5**$_{\mathbf{mst}}$ is generated from the PSS$_{365}$ of an irradiated **M5**$_{\mathbf{st}}$ sample.

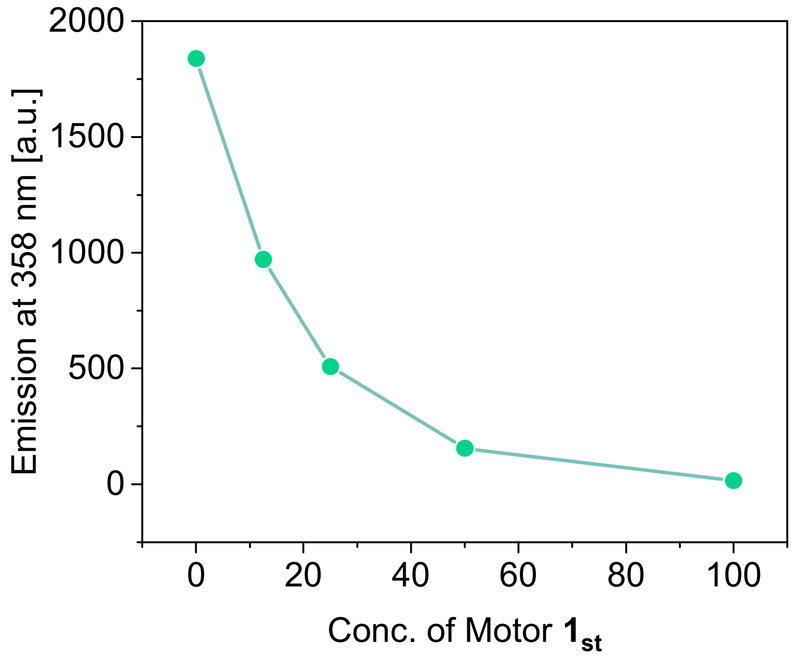

Figure S9. Emission decay of UCNPs upon motor **1**$_{\mathbf{st}}$ addition from 12.5 to 100 uM concentration.

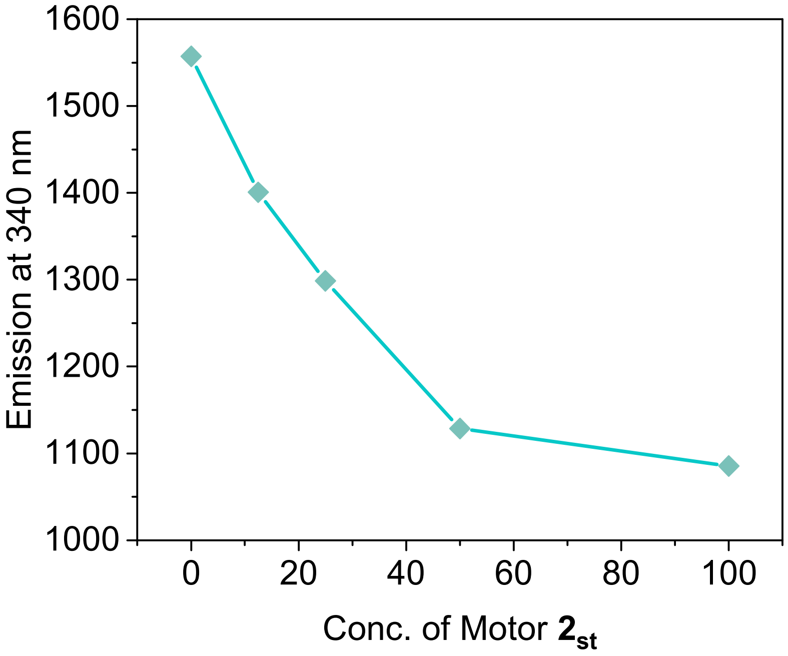

Figure S10. Emission decay of UCNPs upon motor $\mathbf{2}_{st}$ addition from 12.5 to 100 uM concentration.

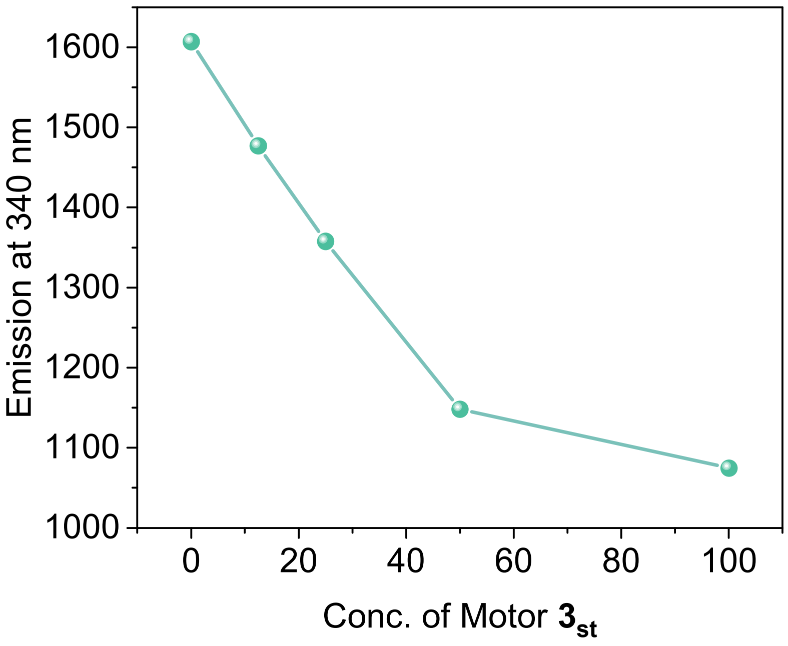

Figure S11. Emission decay of UCNPs upon motor $\mathbf{3}_{st}$ addition from 12.5 to 100 uM concentration.

6. Control UV-Vis experiments

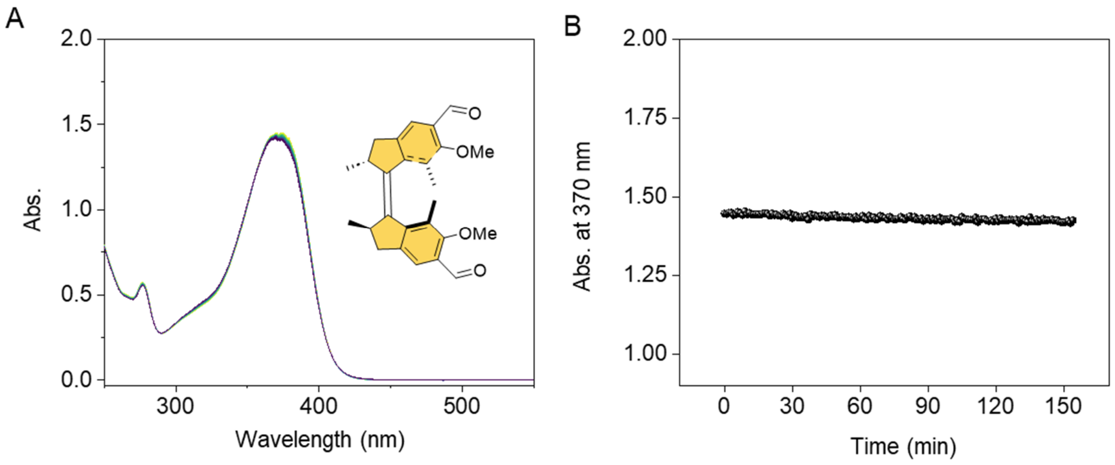

Figure S12. A. UV-Vis spectral changes of Motor $\mathbf{1_{st}}$ (**62** uM, 2 mL, acetonitrile) with 2.5 mg/mL UCNP over 150 min without light irradiation. B. Time decay of Motor $\mathbf{1_{st}}$ absorption at 370 nm.

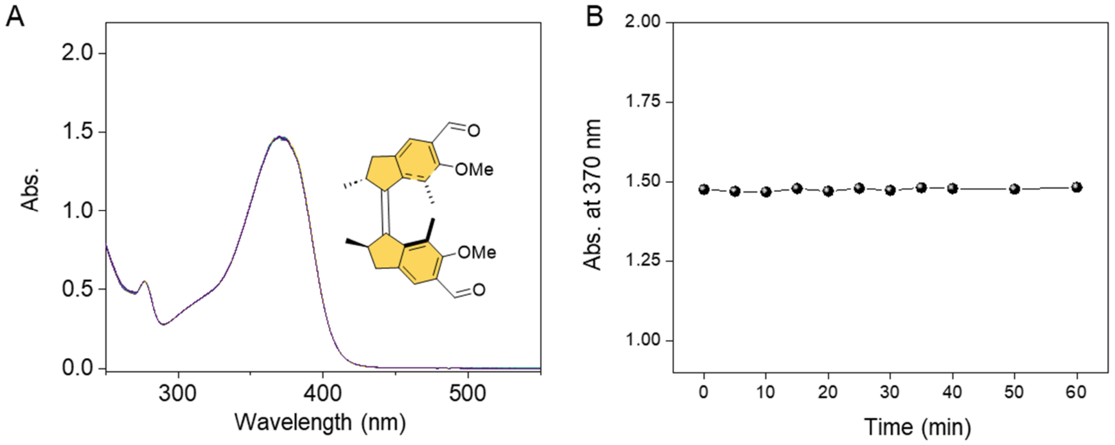

Figure S13. UV-Vis spectral changes of Motor $\mathbf{1_{st}}$ (**62** uM, 2 mL, acetonitrile) with 0 mg/mL UCNP upon irradiation at 980 nm (0.12 W/mm^2) over 60 min. B. Time decay of Motor $\mathbf{1_{st}}$ absorption at 370 nm.

7. UV-Vis studies of NIR light powered photoisomerization of molecular motors.

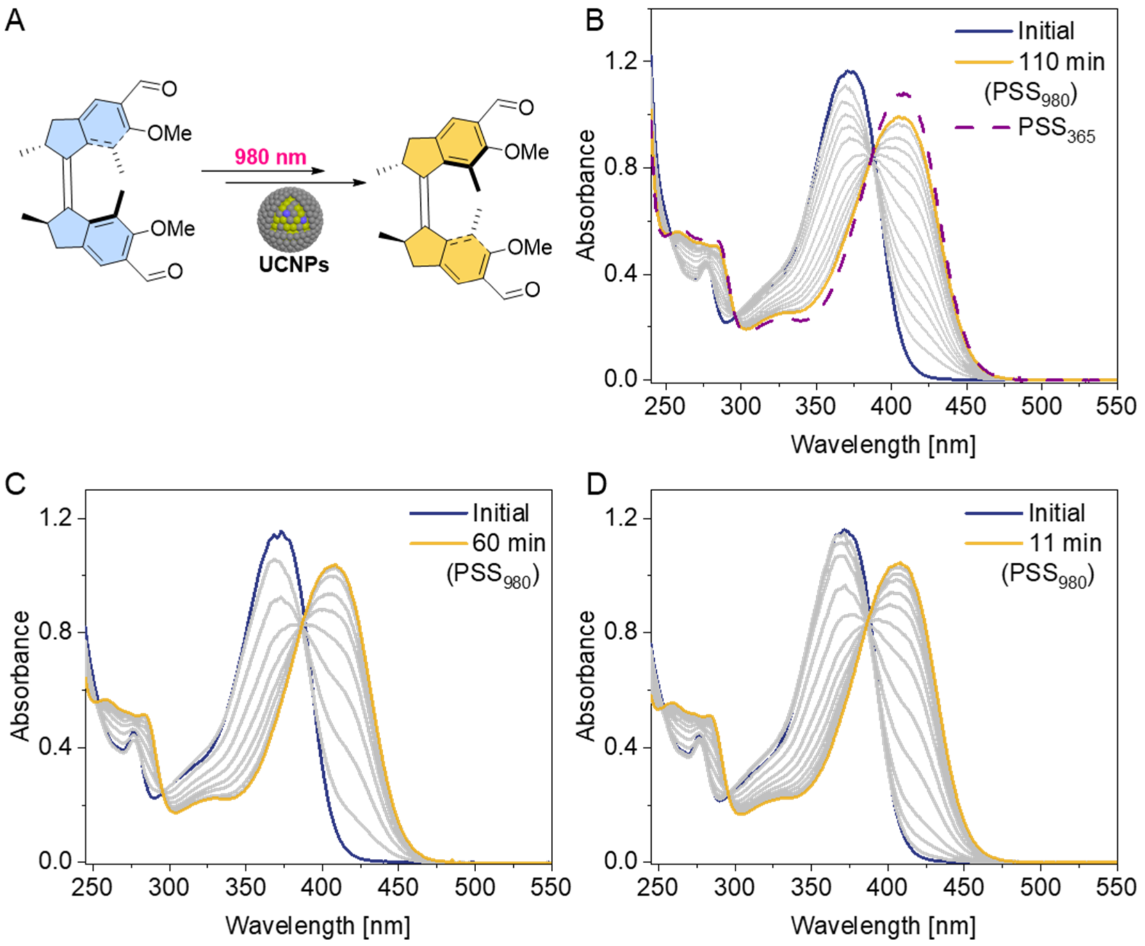

Figure S14. UV-Vis spectral change of motor **1**$_{st}$ (**50** uM, 2 mL, acetonitrile) with **2.5** mg/mL UCNPs upon irradiation at 980 nm with different NIR light power intensity ranging from 0.06 W/mm^2, 0.12 W/mm^2 and 0.24 W/mm^2, from B to D, respectively.

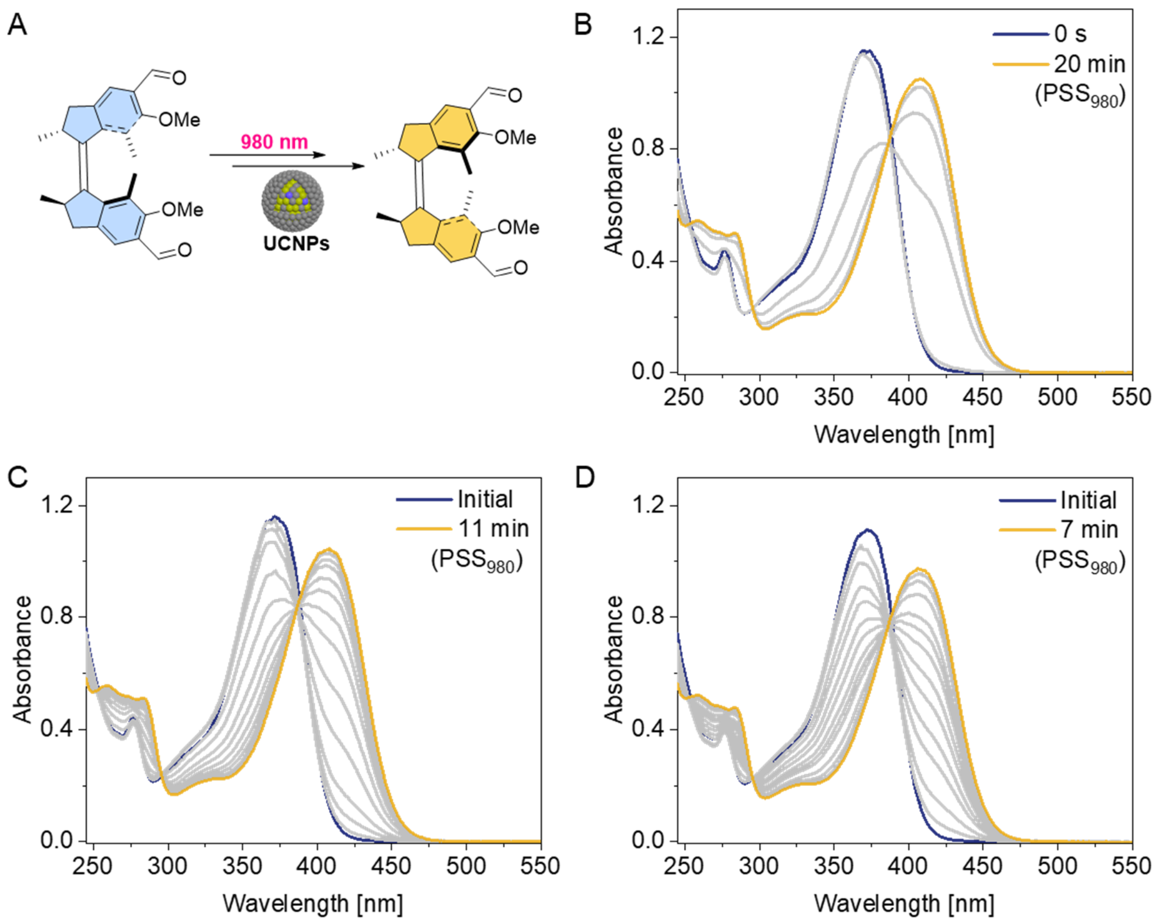

Figure S15. UV-Vis spectral change of motor $\mathbf{1_{st}}$ (**50** uM, in acetonitrile) upon irradiation at 980 nm laser (0.24 W/mm^2), with different concentration of UCNPs ranging from **1.5** mg/mL, **2.5** mg/mL, and **3.5** mg/mL from B to D, respectively.

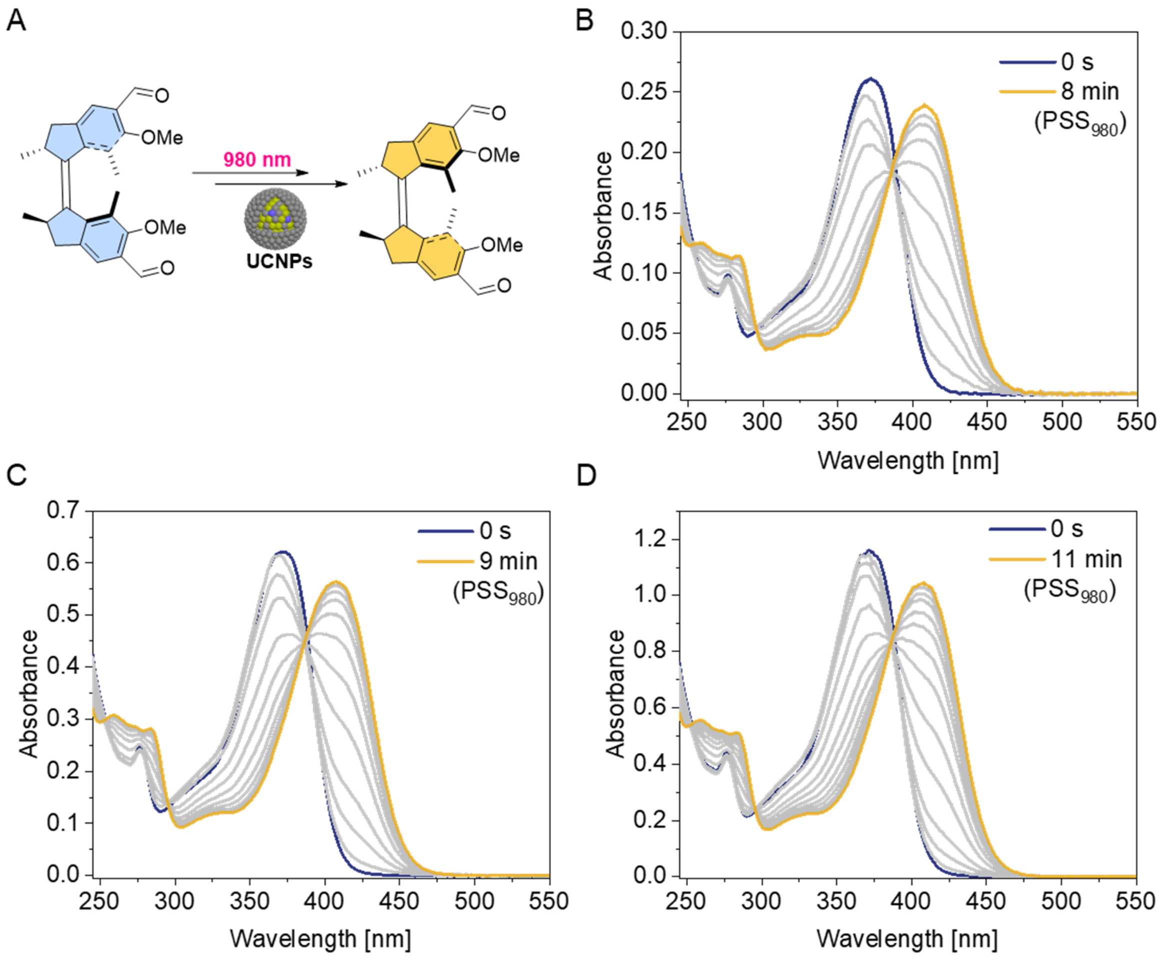

Figure S16. UV-Vis spectral change of motor **1**$_{st}$ (in acetonitrile) with **2.5** mg/mL UCNPs upon irradiation at 980 nm (0.12 W/mm^2). The concentration of Motor **1**$_{st}$ was used from **12** uM, **25** uM and **50** uM from B to D, respectively.

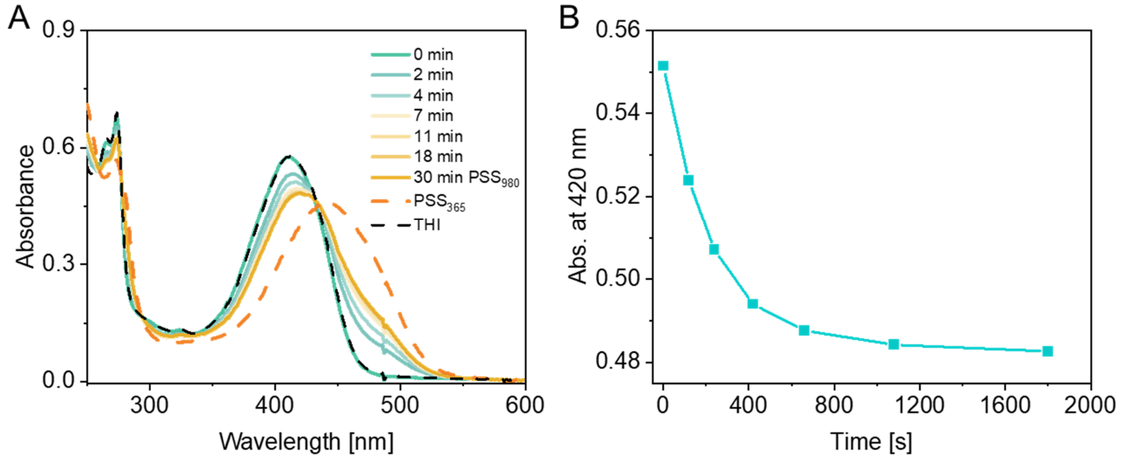

Figure S17. A. UV-vis spectral change of molecular motor **2**$_{st}$ (**50** uM, in acetonitrile, 4 ^{o}C) with UCNPs (**2.5** mg/mL) upon 980 nm laser (0.24 W/mm^2 intensity) or 365 nm LED light irradiation. B. Absorption decay of motor **2**$_{st}$ at 420 nm upon 980 nm laser irradiation.

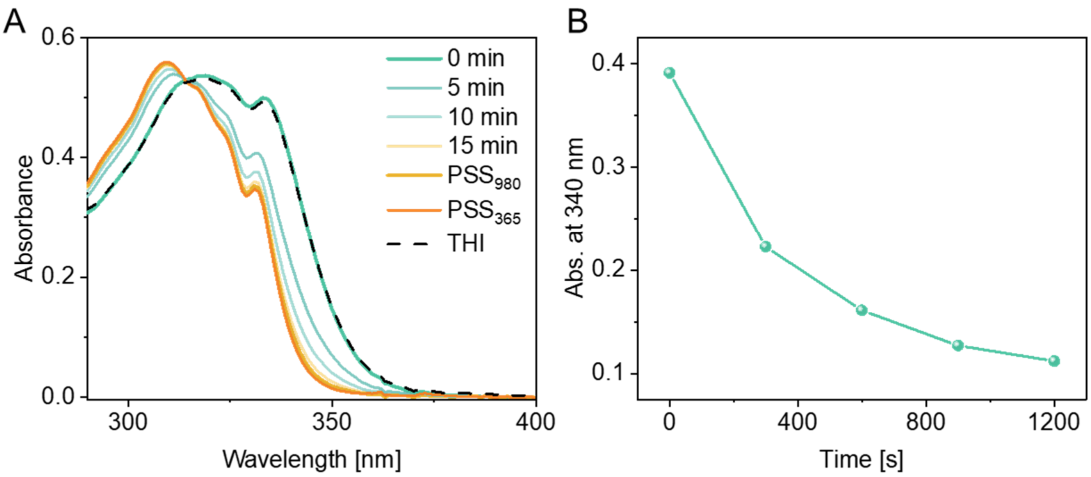

Figure S18. A. UV-Vis spectral change of molecular motor $\mathbf{3_{st}}$ (**50** uM, in acetonitrile, 20 °C) with UCNPs (**2.5** mg/mL) upon 980 nm laser (0.24 W/mm^2 intensity) or 365 nm LED light irradiation. B. Absorption decay of motor $\mathbf{3_{st}}$ at 340 nm upon 980 nm laser irradiation.

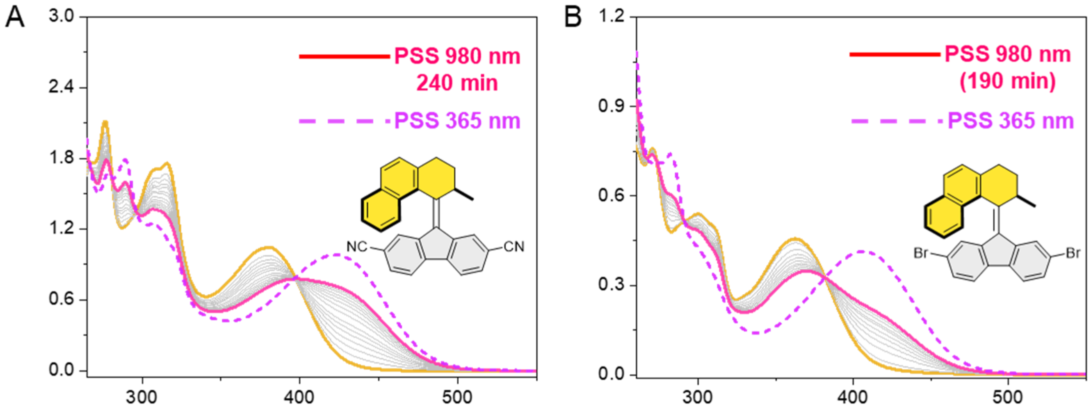

Figure S19. Photoisomerization of overcrowded alkene switches (A) **4** and (B) **5** (**50** uM, in acetonitrile, 20 °C) with **2.5** mg/mL UCNPs by 980 nm laser (0.12 W/mm^2 intensity) or 365 nm LED light irradiation recorded by UV-Vis spectroscopy.

8. Local heating effect investigation induced by NIR absorption.

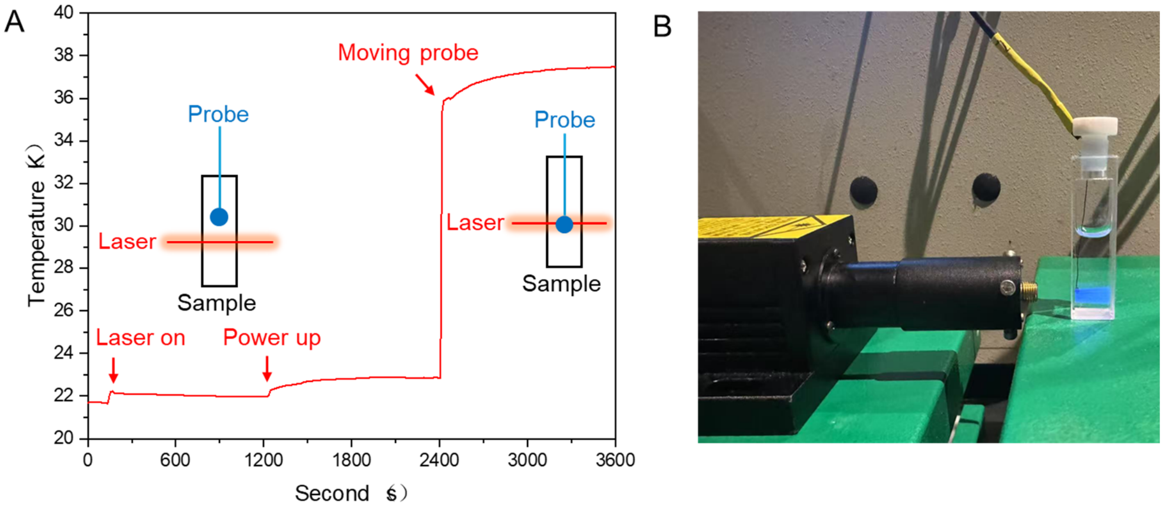

Figure S20 A. Temperature profile of the UCNP–motor dispersion in MeCN under 980 nm laser irradiation. The laser (0.4 W) was turned on at 120 s and increased to 0.6 W at 1200 s. The temperature remained stable (~22 °C) until 2400 s, when the thermal probe was moved into the laser beam path, leading to a rapid temperature rise to ~36 °C. Schematics illustrate the probe position before and after alignment with the beam. B. Experimental setup of local heating measurement.

9 NMR data

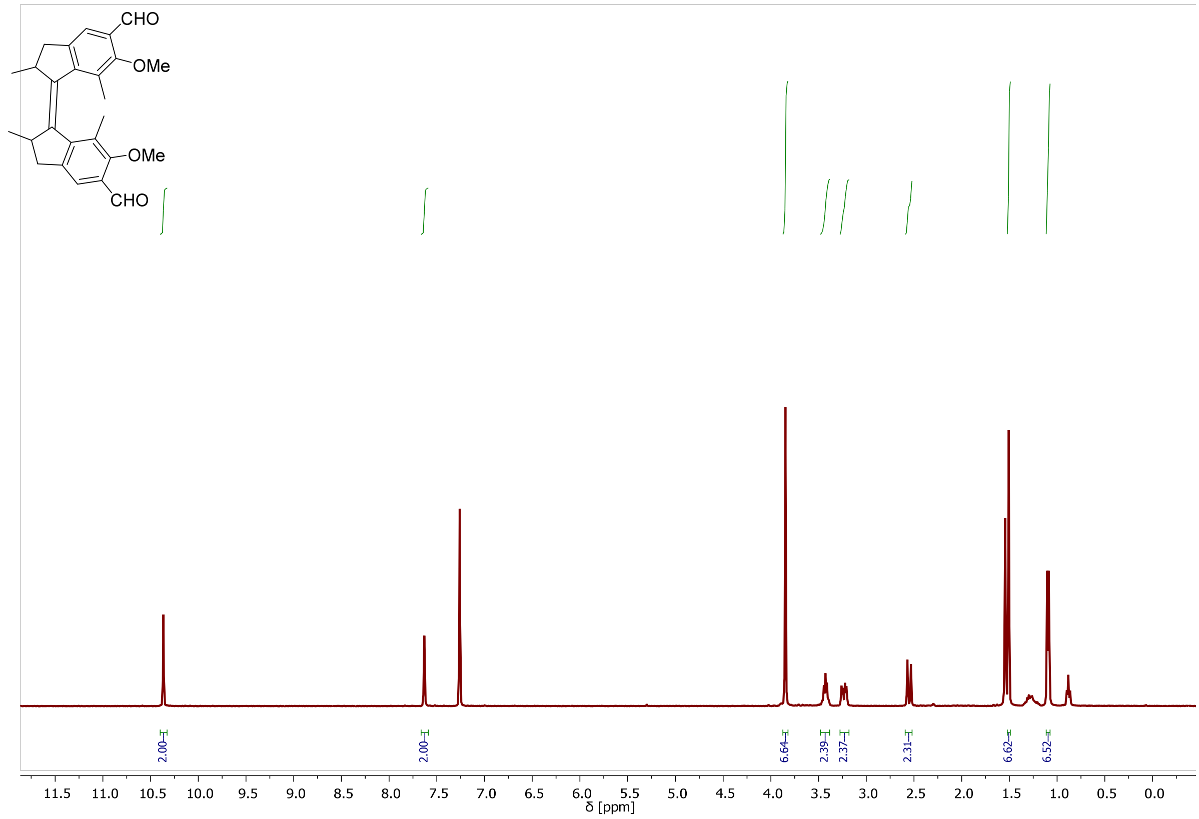

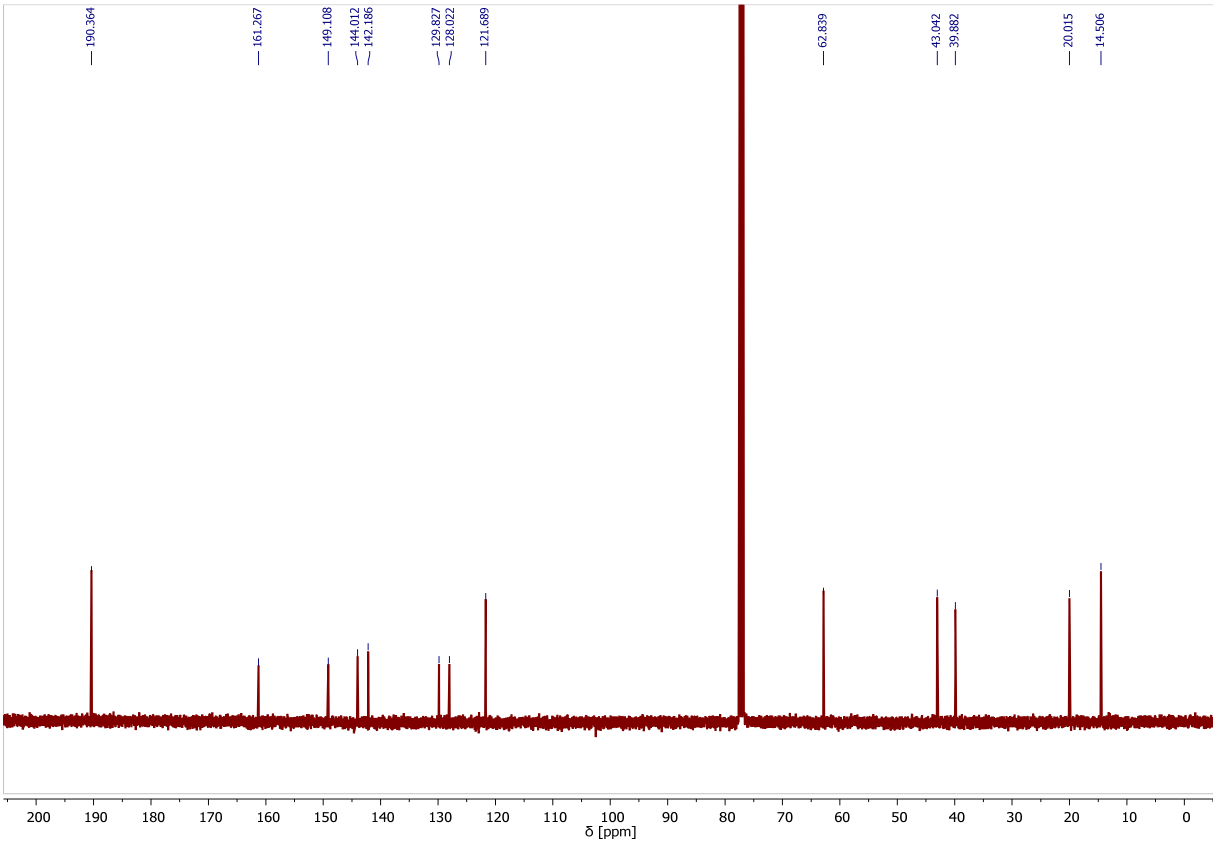

^{1}H NMR (400 MHz, $CDCl_3$) and ^{13}C NMR (101 MHz, $CDCl_3$) spectra of Motor **1**.

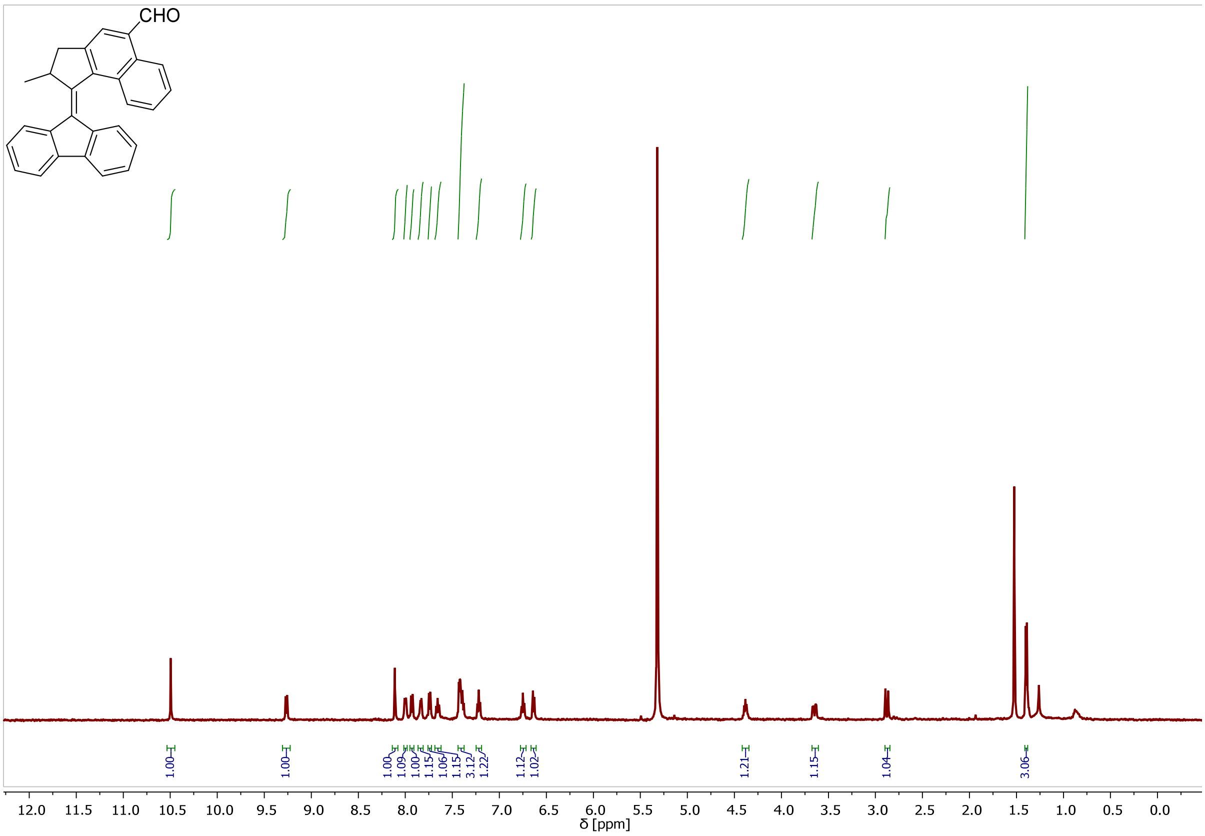

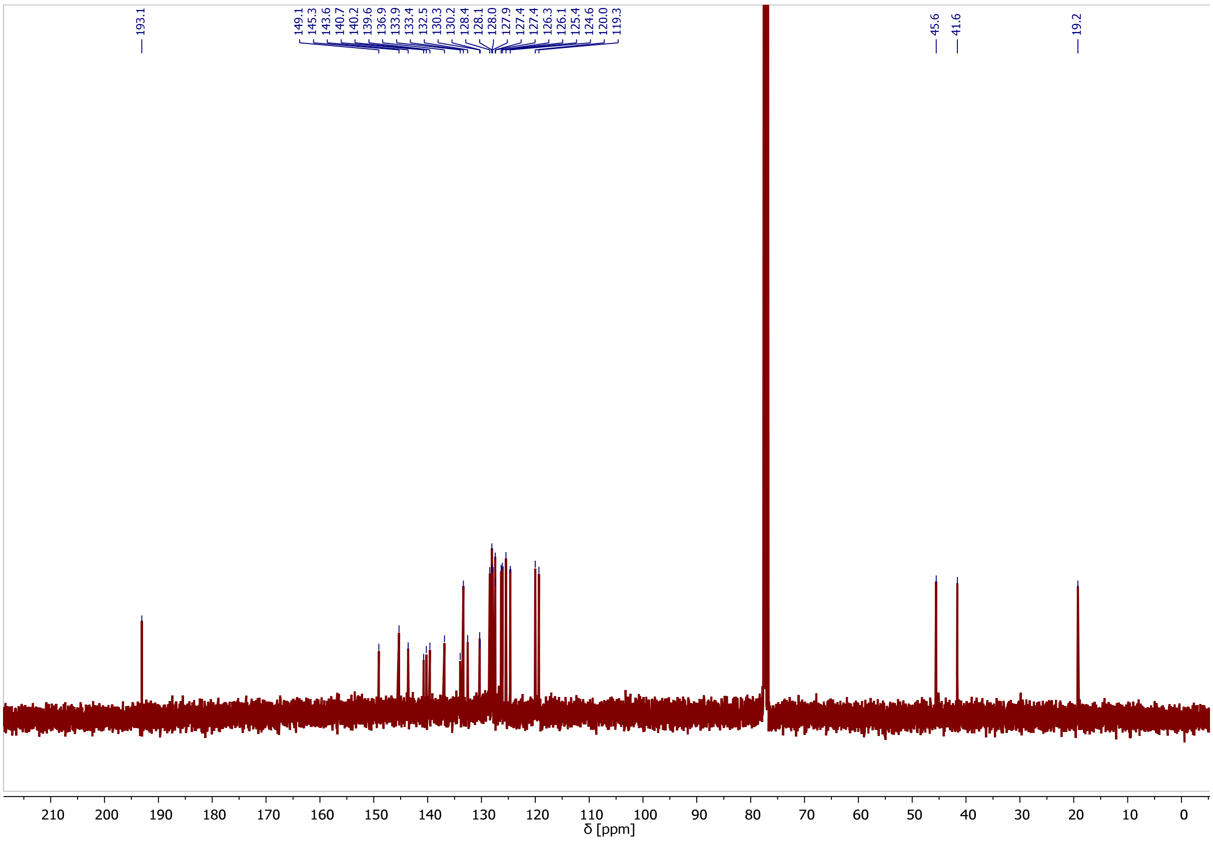

^{1}H (500 MHz, CD_2Cl_2) and ^{13}C NMR (101 MHz, $CDCl_3$) of compound **2.**

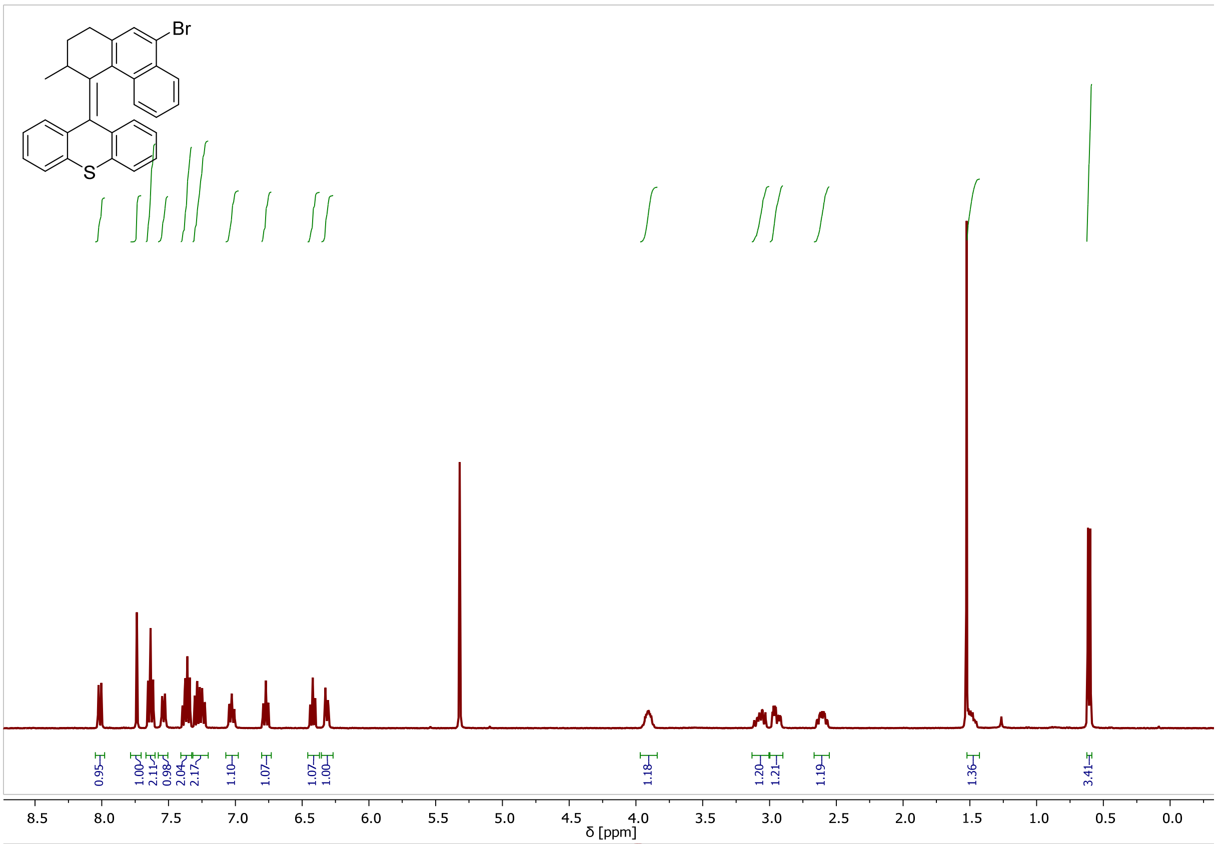

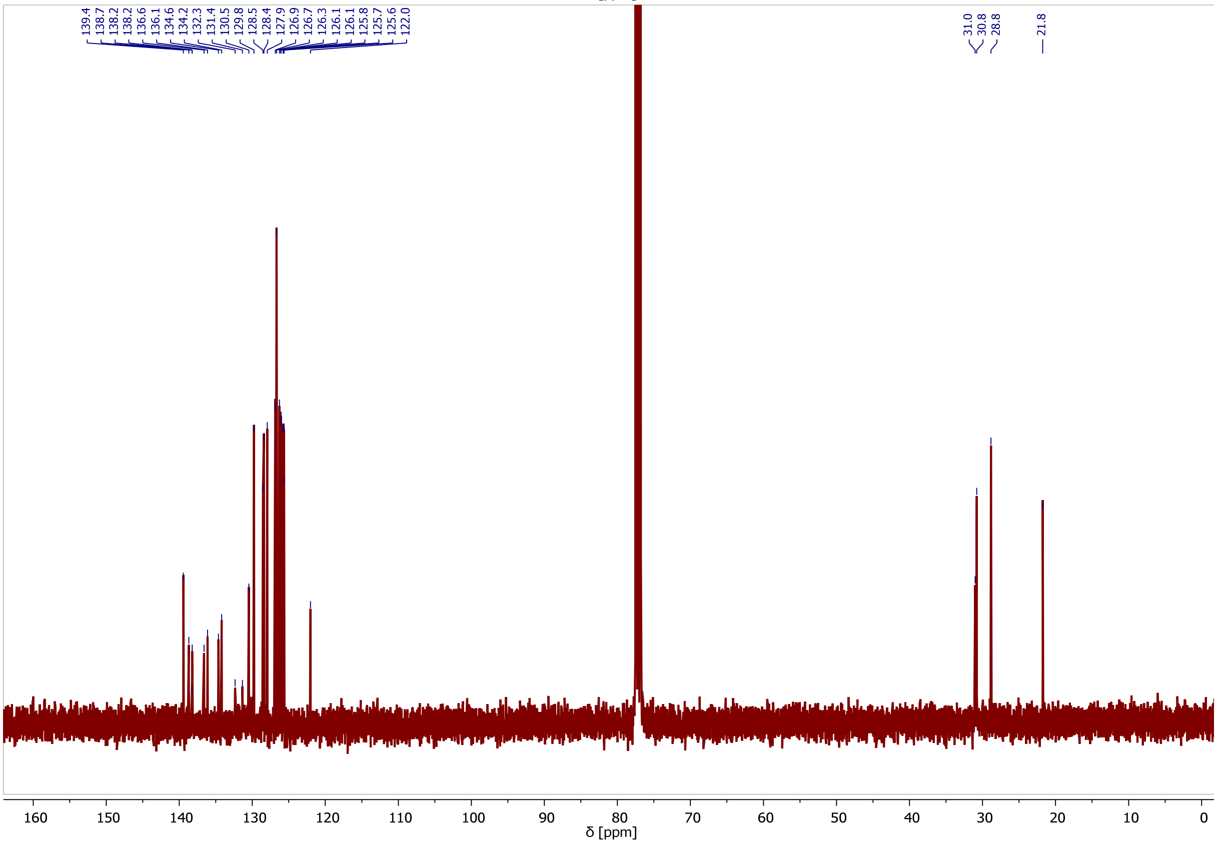

^{1}H NMR (400 MHz, $CDCl_3$) and ^{13}C NMR (101 MHz, $CDCl_3$) spectra of compound **3.**

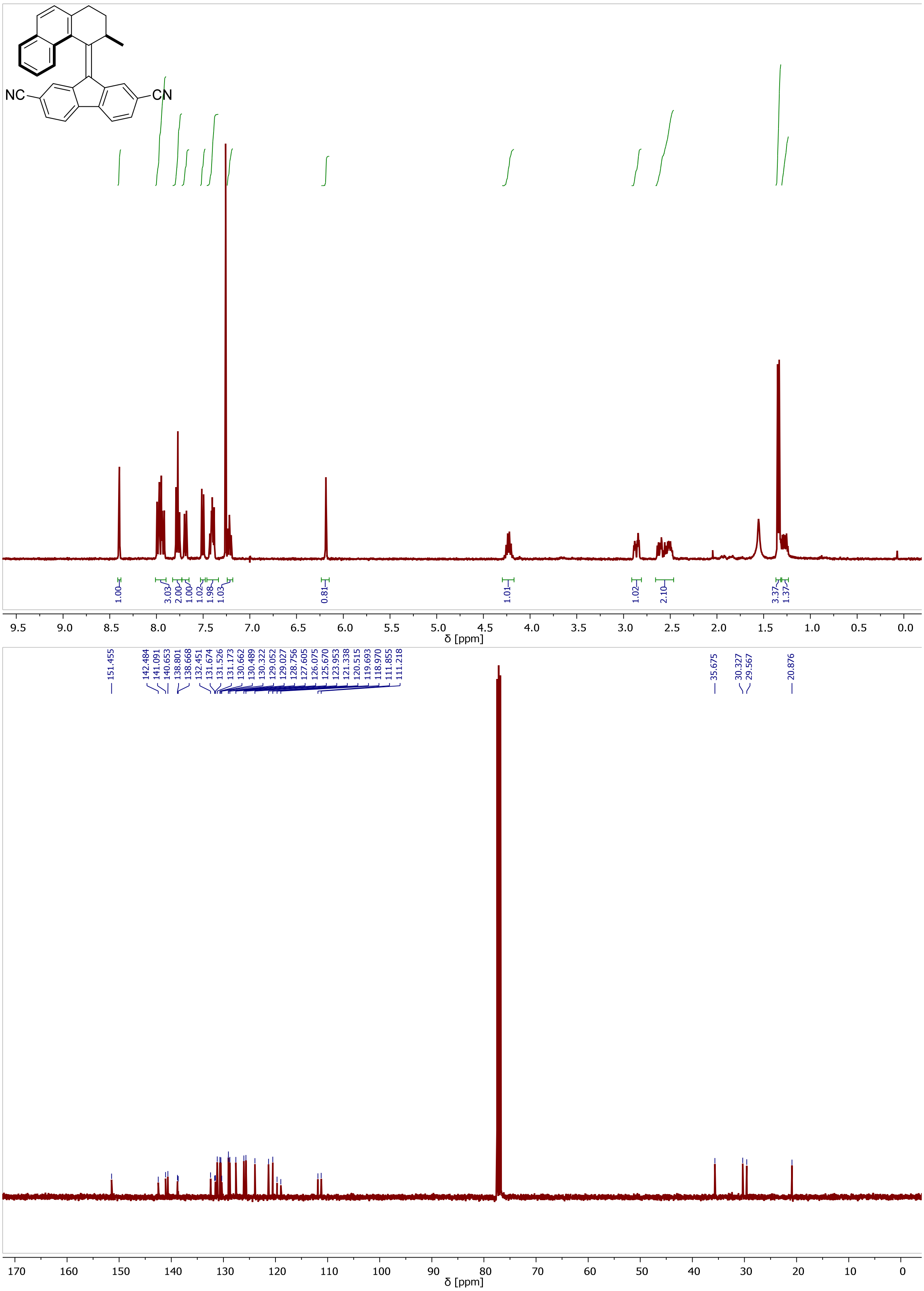

^{1}H NMR (400 MHz, $CDCl_3$) and ^{13}C NMR (101 MHz, $CDCl_3$) spectra of compound **5**.

10. References

1 Li, Z. & Zhang, Y. An Efficient and User-Friendly Method for the Synthesis of Hexagonal-Phase $NaYF_4$:Yb,Er/Tm Nanocrystals with Controllable Shape and Upconversion Fluorescence. Nanotechnology **19**, 345606-345606 (2008).

2 Qian, H. S. & Zhang, Y. Synthesis of hexagonal-phase core-shell $NaYF_4$ nanocrystals with tunable upconversion fluorescence. Langmuir **24**, 12123-12125 (2008).

3 Sheng, J. *et al.* Formylation boosts the performance of light-driven overcrowded alkene-derived rotary molecular motors. *Nat Chem* **16**, 1330-1338 (2024).

4 Sheng, J. *et al.* General strategy for boosting the performance of speed-tunable rotary molecular motors with visible light. *Sci. Adv.*, **11**, eadr9326 (2025).

5 Sheng, J. *et al.* Designing P-type bi-stable overcrowded alkene-based chiroptical photoswitches. *Chem. Sci.*, **14**, 4328-4336 (2023).
