# Supplementary figures and images for: Highly Efficient Near-Infrared Light-Driven Molecular Motor Rotation Enabled by Upconversion Nanoparticles as Nanoscale Light Sources
Source: J Am Chem Soc. 2025 Jul 17;147(30):26797–803. doi: 10.1021/jacs.5c07953 (PMC12314898; doi:10.1021/jacs.5c07953)

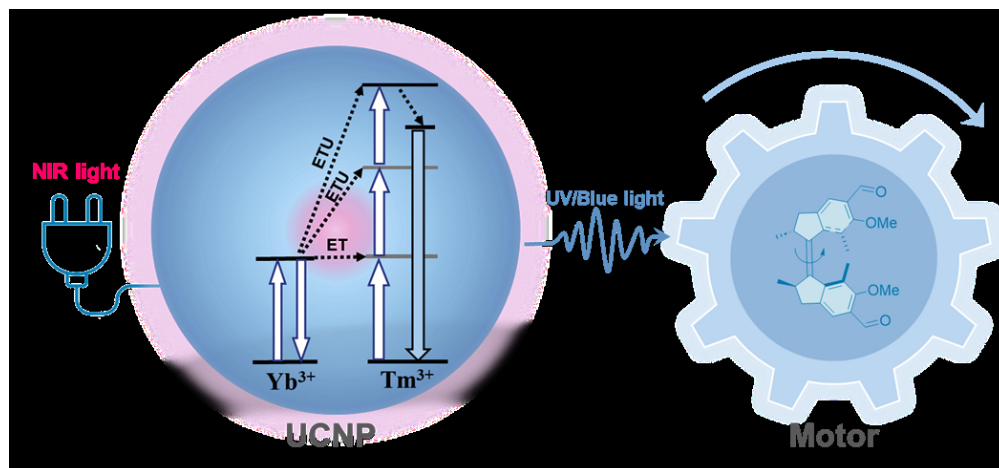

177x82mm (150 x 150 DPI)

Supplement: Supplementary file 2 [file ja5c07953_si_002.pdf]
